# Supplementary material for: Experimental study on engineering properties of fiber-stabilized carbide-slag-solidified soil
Source: PLoS One. 2022 Apr 14;17(4):e0266732. doi: 10.1371/journal.pone.0266732 (PMC9009615; doi:10.1371/journal.pone.0266732)
Supplement: S1 Table — (PDF) [file pone.0266732.s001.pdf]

S1Table Results of Compaction Test

ω （%）	9.24	11.35	13.62	15.03	16.49
ρ_{dmax}（g/cm^3）	1.62	1.64	1.68	1.65	1.60
