# Supplementary material for: Experimental study on engineering properties of fiber-stabilized carbide-slag-solidified soil
Source: PLoS One. 2022 Apr 14;17(4):e0266732. doi: 10.1371/journal.pone.0266732 (PMC9009615; doi:10.1371/journal.pone.0266732)
Supplement: S2 Table — (PDF) [file pone.0266732.s002.pdf]

S2 Table Results of Unconfined Compression Strength Test

Fiber length (mm)	Fiber content (%)	Unconfined compression strength（*MP*a）	
		7d	28d
	0	0.98	1.52
6	0.1	0.99	1.56
	0.2	1.03	1.62
	0.3	1.04	1.73
	0.4	1.04	1.67
12	0.1	1.01	1.59
	0.2	1.03	1.63
	0.3	1.04	1.66
	0.4	1.06	1.67
19	0.1	1.02	1.62
	0.2	1.03	1.64
	0.3	1.05	1.69
	0.4	1.06	1.72
