# Supplementary material for: Experimental study on engineering properties of fiber-stabilized carbide-slag-solidified soil
Source: PLoS One. 2022 Apr 14;17(4):e0266732. doi: 10.1371/journal.pone.0266732 (PMC9009615; doi:10.1371/journal.pone.0266732)
Supplement: S3 Table — (PDF) [file pone.0266732.s003.pdf]

S3 Table Results of Indirect Tensile Strength Test

Fiber length (mm)	Fiber content (%)	Indirect tensile strength （MPa）	
		7d	28d
	0	0.065	0.107
6	0.1	0.068	0.139
	0.2	0.067	0.142
	0.3	0.070	0.156
	0.4	0.071	0.151
12	0.1	0.069	0.154
	0.2	0.070	0.167
	0.3	0.071	0.179
	0.4	0.073	0.176
19	0.1	0.070	0.164
	0.2	0.072	0.177
	0.3	0.075	0.189
	0.4	0.072	0.187
