# Supplementary material for: Experimental study on engineering properties of fiber-stabilized carbide-slag-solidified soil
Source: PLoS One. 2022 Apr 14;17(4):e0266732. doi: 10.1371/journal.pone.0266732 (PMC9009615; doi:10.1371/journal.pone.0266732)
Supplement: Attachment — Submitted filename: PONE-D-22-00217_R1.pdf [file pone.0266732.s007.pdf]

PLOS ONE

Experimental study on engineering properties of fiber-stabilized carbide-slag-solidified soil

--Manuscript Draft--

**Manuscript Number:**	PONE-D-22-00217R1
**Article Type:**	Research Article
**Full Title:**	Experimental study on engineering properties of fiber-stabilized carbide-slag-solidified soil
**Short Title:**	Fiber-stabilized carbide-slag-solidified soil
**Corresponding Author:**	zhang hongzhou<br>China University of Geosciences Beijing<br>Beijing, CHINA
**Keywords:**	fiber; carbide slag; unconfined compressive strength; indirect tensile strength; solidified soil
**Abstract:**	Carbide slag has been used to prepare solidified soil to effectively reduce the stacking and disposal of carbide slag and achieve efficient resource utilization. Because of the significant brittleness and low strength of carbide-slag-stabilized soil, fibers were added to carbide-slag-stabilized soil in this experimental study. The effects of fiber length and fiber content on the unconfined compressive and indirect tensile strengths of carbide-slag-stabilized soil were investigated. The concepts of the density of fibers in solidified soil and the number of fibers in a unit volume solidified soil were proposed, and the effects of fiber distribution density on the mechanical properties of the solidified soil were evaluated. The fibers increased the indirect tensile strength of the carbide-slag-solidified soil, which was significantly higher than the unconfined compressive strength of the solidified soil. The fibers had no significant effect on the unconfined compressive and indirect tensile strengths of the 7 d carbide-slag-solidified soil but increased those of the 28 d carbide-slag-solidified soil. The enhancement effect was the most significant when a 0.3% content of 19 mm long fibers was incorporated into the carbide-slag-solidified soil.
**Order of Authors:**	zhang hongzhou
	Tian Limei
	Wang Shuang
	Qiao Yanhong
**Response to Reviewers:**	Dear Reviewers:<br>We tried our best to improve the manuscript and made some changes in the manuscript. These changes will not influence the content and framework of the paper. And here we did not list the changes but marked in red in revised paper.<br>We appreciate for Reviewers' warm work earnestly, and hope that the correction will meet with approval. Once again, thank you very much for your comments and suggestions.<br>Thank you and best regards.<br>Yours sincerely,<br>Hongzhou Zhang.
**Additional Information:**	
**Question**	**Response**
**Financial Disclosure**<br><br>Enter a financial disclosure statement that describes the sources of funding for the work included in this submission. Review the submission guidelines for detailed	This study was funded by Science and Technology Support Project of Langfang(Grant Number 2021013167)，Science and Technology Support Project of Langfang Normal University(Grant Number XBQ202113).

requirements. View published research articles from *PLOS ONE* for specific examples.<br><br>This statement is required for submission and **will appear in the published article** if the submission is accepted. Please make sure it is accurate.<br><br>**Unfunded studies**<br>Enter: *The author(s) received no specific funding for this work.*<br><br>**Funded studies**<br>Enter a statement with the following details:<br>• Initials of the authors who received each award<br>• Grant numbers awarded to each author<br>• The full name of each funder<br>• URL of each funder website<br>• Did the sponsors or funders play any role in the study design, data collection and analysis, decision to publish, or preparation of the manuscript?<br>• **NO** - Include this sentence at the end of your statement: *The funders had no role in study design, data collection and analysis, decision to publish, or preparation of the manuscript.*<br>• **YES** - Specify the role(s) played.<br><br>* typeset	
**Competing Interests**<br><br>Use the instructions below to enter a competing interest statement for this submission. On behalf of all authors, disclose any competing interests that could be perceived to bias this work—acknowledging all financial support and any other relevant financial or non-financial competing interests.<br><br>This statement is required for submission and **will appear in the published article** if the submission is accepted. Please make sure it is accurate and that any funding sources listed in your Funding Information later in the submission form are also declared in your Financial Disclosure	The authors have declared that no competing interests exist.

statement.<br><br>View published research articles from *PLOS ONE* for specific examples.<br><br>**NO authors have competing interests**<br><br>Enter: *The authors have declared that no competing interests exist.*<br><br>**Authors with competing interests**<br><br>Enter competing interest details beginning with this statement:<br><br>*I have read the journal's policy and the authors of this manuscript have the following competing interests: [insert competing interests here]*<br><br>* typeset	
**Ethics Statement**<br><br>Enter an ethics statement for this submission. This statement is required if the study involved:<br><br>• Human participants<br>• Human specimens or tissue<br>• Vertebrate animals or cephalopods<br>• Vertebrate embryos or tissues<br>• Field research<br><br>Write "N/A" if the submission does not require an ethics statement.<br><br>General guidance is provided below. Consult the submission guidelines for detailed instructions. **Make sure that all information entered here is included in the Methods section of the manuscript.**	N

**Format for specific study types**<br><br>**Human Subject Research (involving human participants and/or tissue)**<br>• Give the name of the institutional review board or ethics committee that approved the study<br>• Include the approval number and/or a statement indicating approval of this research<br>• Indicate the form of consent obtained (written/oral) or the reason that consent was not obtained (e.g. the data were analyzed anonymously)<br><br>**Animal Research (involving vertebrate animals, embryos or tissues)**<br>• Provide the name of the Institutional Animal Care and Use Committee (IACUC) or other relevant ethics board that reviewed the study protocol, and indicate whether they approved this research or granted a formal waiver of ethical approval<br>• Include an approval number if one was obtained<br>• If the study involved *non-human primates*, add *additional details* about animal welfare and steps taken to ameliorate suffering<br>• If anesthesia, euthanasia, or any kind of animal sacrifice is part of the study, include briefly which substances and/or methods were applied<br><br>**Field Research**<br><br>Include the following details if this study involves the collection of plant, animal, or other materials from a natural setting:<br>• Field permit number<br>• Name of the institution or relevant body that granted permission	
**Data Availability**<br><br>Authors are required to make all data underlying the findings described fully available, without restriction, and from the time of publication. PLOS allows rare exceptions to address legal and ethical concerns. See the PLOS Data Policy and FAQ for detailed information.	Yes - all data are fully available without restriction

Powered by Editorial Manager® and ProduXion Manager® from Aries Systems Corporation

A Data Availability Statement describing where the data can be found is required at submission. Your answers to this question constitute the Data Availability Statement and **will be published in the article**, if accepted.<br><br>**Important:** Stating 'data available on request from the author' is not sufficient. If your data are only available upon request, select 'No' for the first question and explain your exceptional situation in the text box.<br><br>Do the authors confirm that all data underlying the findings described in their manuscript are fully available without restriction?	
**Describe where the data may be found in full sentences. If you are copying our sample text, replace any instances of XXX with the appropriate details.**<br><br>• If the data are **held or will be held in a public repository**, include URLs, accession numbers or DOIs. If this information will only be available after acceptance, indicate this by ticking the box below. For example: *All XXX files are available from the XXX database (accession number(s) XXX, XXX.).*<br>• If the data are all contained **within the manuscript and/or Supporting Information files**, enter the following: *All relevant data are within the manuscript and its Supporting Information files.*<br>• If neither of these applies but you are able to provide **details of access elsewhere**, with or without limitations, please do so. For example:<br><br>*Data cannot be shared publicly because of [XXX]. Data are available from the XXX Institutional Data Access / Ethics Committee (contact via XXX) for researchers who meet the criteria for access to confidential data.*<br><br>*The data underlying the results presented in the study are available from (include the name of the third party*	All relevant data are within the manuscript and its Supporting Information files.

and contact information or URL).<br>• This text is appropriate if the data are owned by a third party and authors do not have permission to share the data.<br><br>* typeset	
Additional data availability information:	Tick here if the URLs/accession numbers/DOIs will be available only after acceptance of the manuscript for publication so that we can ensure their inclusion before publication.; Tick here if your circumstances are not covered by the questions above and you need the journal's help to make your data available.

Powered by Editorial Manager® and ProduXion Manager® from Aries Systems Corporation

Manuscript Click here to access/download;Manuscript;Manuscript.docx

Experimental study on engineering properties of fiber-stabilized carbide-slag-solidified soil

Hongzhou Zhang[1,2*], Limei Tian[2], Shuang Wang[2], Yanhong Qiao[2]

[1] China University of Geosciences Beijing, Beijing, China

[2] Langfang Normal University, Langfang, China

[*] Corresponding author

E-mail: zhanghongzhou@lfnu.edu.cn

Abstract

Carbide slag has been used to prepare solidified soil to effectively reduce the stacking and disposal of carbide slag and achieve efficient resource utilization. Because of the significant brittleness and low strength of carbide-slag-stabilized soil, fibers were added to carbide-slag-stabilized soil in this experimental study. The effects of fiber length and fiber content on the unconfined compressive and indirect tensile strengths of carbide-slag-stabilized soil were investigated. The concepts of the density of fibers in solidified soil and the number of fibers in a unit volume solidified soil were proposed, and the effects of fiber distribution density on the mechanical properties of the solidified soil were evaluated. The fibers increased the indirect tensile strength of the carbide-slag-solidified soil, which was significantly higher than the unconfined compressive strength of the solidified soil. The fibers had no significant effect on the unconfined compressive and indirect tensile strengths of

the 7 d carbide-slag-solidified soil but increased those of the 28 d carbide-slag-solidified soil. The enhancement effect was the most significant when a 0.3% content of 19 mm long fibers was incorporated into the carbide-slag-solidified soil.

Keywords

fiber; carbide slag; unconfined compressive strength; indirect tensile strength; solidified soil

Introduction

The efficient treatment and utilization of carbide slag is the only approach to solve the environmental pollution problem of the carbide industry and achieve sustainable development. Many researchers have attempted to utilize calcium carbide slag for soil reinforcement [1–3], and the solidified soil has problems, such as low strength and toughness and susceptibility to cracking [4]. After more than four wetting–drying cycles, the compressive strength of the carbide-slag-stabilized soil fails to satisfy the strength requirements of the subgrade [5,6].

Using fibers to stabilize soil is an effective soil reinforcement technique. Fibers can significantly increase the strength and toughness of soil and solve soil cracking problems [7,8]. The addition of fibers effectively improves the tensile strain and reduces the cracking of specimens [9]. Artificial and natural fibers significantly improve the residual shear strength and unconfined compressive strength limits of soil [10]. Polypropylene fibers can improve the compressive strength, shear strength, and deformation resistance of cement-clay [11–13]. The incorporation of fibers had obvious effect on delaying the cracking of carbide slag stabilized soil specimens under load. [14]. The reinforcement effect of the fiber depends on the strength of the interface between the reinforcement and soil, and the mechanical interactions between the reinforcement and soil are

mainly adhesion and friction [15]. The stability mechanism of fibers in the soil can be summarized as “bending mechanism” and “intercrossing mechanism” [16]. The reinforcement effect of fibers in the soil is anisotropic [17]. When the orientation angle of a fiber is perpendicular to the direction of the strong contact force, the fiber exerts the maximum effect [18]. Fibers can improve the ductility and compressive resistance of reinforced soil and reduce the sensitivity of soil to water [19]. Currently, research on the effects of fiber length and fiber content on the properties of fiber-stabilized carbide-slag-solidified soil has not been conducted extensively.

In this study, fiber-stabilized carbide-slag-solidified silt was adopted as the research object. Based on unconfined compressive strength and indirect tensile strength tests, the effects of fiber length and fiber content on the performance of carbide-slag-solidified silt was investigated, and the optimum fiber length and content were determined. The concepts of the mass density of fibers in the solidified soil (DFS) and the root number of fibers in a unit volume solidified soil (NFS) were proposed. Moreover, the effects of fiber distribution density on the mechanical properties of the solidified soil were evaluated.

In this study, the properties of fiber-carbide slag-solidified silt were studied according to fiber length and fiber content. The research findings are expected to serve as a reference for applying fiber-stabilized carbide-slag-solidified soil in road engineering. It can protect the environment, improve the resource utilization efficiency of carbide slag, reduce the project cost, achieve sustainable development, and achieve remarkable economic and social benefits.

Materials and methods

Materials

The soil samples used for the tests were collected from the Airport, New Area of Langfang, Hebei Province, China. The study did not involve private or protected land. According to Test Methods of soils for Highway Engineering (JTG 3430-2020) [20], the physical indexes and particle gradation of test soil are determined. The physical indexes are listed in Table 1, and the particle size distribution is shown in Fig 1, where C_u and C_c are 13 and 1.06, respectively. Before the tests, the soil samples were crushed by soil milling and passed through a 2.36 mm square hole sieve. The soil collected under the sieve was used for testing.

Table 1. Physical Indexes of Soil Used for Testing.

**Property**	ω_L	ω_P	I_P	G	σ_{dmax}	OMC
	**%**	**%**	**%**		**g/cm^3**	**%**
**Value**	31.1	22.8	8.3	2.45	1.86	12.15

Carbide slag was purchased from Dezhou Shihua Chemical Co., Ltd. The raw calcium carbide slag had a high moisture content and a peculiar smell. After drying and dehydration, it was crushed by soil milling and passed through a 0.075 mm square hole sieve. The carbide slag under the sieve was used for the tests (Fig 2). The main components of the calcium carbide slag are listed in Table 2. The carbide slag contained a large amount of calcium oxide similar to that of quick lime and exhibited significant activity (Table 2).

Table 2. Chemical Composition of Carbide Slag.

**Chemical compound**	**CaO**	**SiO_2**	**Al_2O_3**	**Fe_2O_3**	**MgO**	**Loss on ignition**
**Content (%)**	65.77	3.15	1.78	0.24	0.14	23.56

The fibers were purchased from Changsha Ninxiang Building Materials Co., Ltd. Polypropylene fibers with lengths of 6, 12, and 19 mm were used for testing (Fig 3). According to

Testing Method for Length of Man-made Staple Fibers(GB/T 14336-2008) [21] and Testing Method for Tensile Properties of Man-made Staple Fibers(GB/T 14337-2008) [22]，the physical and mechanical properties of the fibers are listed in Table 3.

Table 3. Physical and Mechanical Properties of Polypropylene Fibers.

**Length (mm)**	**Diameter (μm)**	**Density (g/cm^3)**	**Tensile strength (*MP*a)**	***E* (*MP*a)**	**Ultimate elongation (%)**	**Melting point (°C)**
6/12/19	32.7	0.91	469	4236	28.4	169

Methods

This study was divided into three steps to investigate the effects of the fiber length and content on the performance of fiber-stabilized carbide-slag-solidified silt, optimal fiber length and content, and effect of fiber distribution density on the mechanical properties of the solidified soil.

(1) Compaction tests. The optimum moisture content and the maximum dry density of carbide-slag-solidified silt were determined according to “Compaction Test Method for Inorganic Binder Stabilized Materials” (T0804-1994).

(2) Unconfined compressive strength tests. The unconfined compressive strength of fiber-stabilized carbide-slag-solidified silt was determined according to the “Unconfined Compressive Strength Test Method for Inorganic Binder Stabilized Materials” (T0805-1994).

(3) Indirect tensile strength test. The indirect tensile strength of fiber-stabilized calcium-carbide-slag-solidified silt was determined according to “Indirect Tensile Strength Test Method for Inorganic Binder Stabilized Materials (Splitting Test)” (JTGE51-2009-T0806).

Preparation of specimens

The specimens were prepared using the method specified in "Inorganic Binder-Stabilized Material Specimen Production Method (Cylindrical)" (T0843-2009). Dried sieved carbide slag and silt were thoroughly blended at a mass ratio of 2:8. An appropriate amount of the fiber was proportionally added to the mixture and thoroughly stirred manually to fully disperse the fiber into the mixture. An appropriate amount of water was added to the mixture blended with the fiber, stirred evenly, and placed in a plastic bag. The bag was sealed and placed in the dark for 24 h. A 50 mm × 50 mm test mold was adopted, and the calculated weight of the soil sample was determined based on the degree of compaction (96%). Twelve samples were prepared for each group. After the specimens were produced using the static pressure method, they were placed in a curing box (temperature = 20±2 °C; relative humidity ≥ 95%). The specimens are shown in Fig 4. The proportions of the specimens of fiber-stabilized carbide-slag-solidified soil are listed in Table 4.

Table 4. Proportions of Specimens.

Fiber length (mm)	Fiber content (%)	Curing period (d)	Parameter
6	0/0.1/0.2/0.3/0.4	7/28	Unconfined compressive strength test<br>Indirect tensile strength test
12	0.1/0.2/0.3/0.4	7/28	Unconfined compressive strength test<br>Indirect tensile strength test
19	0.1/0.2/0.3/0.4	7/28	Unconfined compressive strength test<br>Indirect tensile strength test

Results

Compaction

According to the experiment, the optimum moisture content of 2:8 carbide-slag-solidified soil was 13%-14%. First, five portions of 2:8 carbide-slag-solidified soil were prepared, and the target moisture contents were 10%, 12%, 14%, 16%, and 18%. Compaction tests were performed according to the compaction test method for inorganic binder stabilization materials (T0804-1994). The compaction curve of the soil samples is shown in (Fig 5). The optimum moisture content of 2:8 carbide-slag-solidified soil was 13.62 %, and the maximum dry density was 1.68g/cm^3. Because the amount of fiber was small, its effects on the optimum moisture content and maximum dry density were not evident. Therefore, when preparing an unconfined compressive strength test and indirect tensile strength test samples, it could be directly configured according to the optimum water content of 13.62 % and the maximum dry density of 1.68g/cm^3.

Unconfined compressive strength

The failure pattern of the specimens subjected to unconfined compression is shown in Fig 6. The unconfined compressive strength test results of 2:8 carbide-slag-solidified soil with different fiber lengths and contents at 7 and 28 d are shown in Fig 7.

The incorporation of fibers somewhat improved the unconfined compressive strength of the solidified soil, but the improvement was limited. For the same fiber length and content, the unconfined compressive strength of the solidified soil significantly increased with increasing curing time. For each group of fibers, the 7 and 28 d unconfined compressive strength increased with increasing fiber content, but the degree of improvement was limited. When the fiber contents were 0.3% and 0.4%, the variation in the unconfined compressive strength was insignificant. For the

solidified soil reinforced with a 6 mm fiber at a 0.3% fiber content, the maximum values of the 7 and 28 d unconfined compressive strengths were 1.04 and 1.67 MPa, respectively. For the solidified soil mixed with a 12 mm fiber, the maximum value of the 7 d unconfined compressive strength (1.06 MPa) was observed at the 0.4% fiber content. The maximum unconfined compressive strength at 28 d was 1.69 MPa when the fiber content was 0.3%. For the solidified soil mixed with a 19 mm fiber, the maximum unconfined compressive strengths at 7 and 28 d were 1.06 and 1.72 MPa, respectively, when the fiber content was 0.4%. With an increase in the fiber length, the unconfined compressive strength of the solidified soil increased slightly, but the degree of increase was minimal.

Indirect tensile strength

The failure patterns of the test specimens subjected to indirect tensile strength test is shown in Fig 8. The indirect tensile strength test results for the calcium-carbide-slag-solidified soil at 7 and 28 d in 2:8 ratio with different fiber lengths and contents are presented in Fig 9. The addition of fiber did not influence the indirect tensile strength of the solidified soil at 7 d but significantly increased the indirect tensile strength at 28 d (Fig 9). For the same fiber length and content, the indirect tensile strength of the solidified soil significantly increased with increasing curing time. With an increase in the fiber length for the same fiber content, the indirect tensile strength of the 7 d solidified soil did not increase significantly; in contrast, the indirect tensile strength of the 28 d solidified soil increased significantly. When the fiber length was 6 mm, and the fiber content was 0.4%, the maximum indirect tensile strength at 7 d was 0.071 MPa. The maximum indirect tensile strength at 28 d was 0.156 MPa when the fiber content was 0.3%. When the fiber length was 12 mm

using a fiber content of 0.3%, the maximum indirect tensile strength at 28 d was 0.156 MPa. The maximum indirect tensile strength of the 7 d cured soil was 0.073 MPa when the fiber content was 0.4%. The maximum indirect tensile strength of the cured soil at 28 d was 0.179 MPa when the fiber content was 0.3%. The maximum indirect tensile strength of the 7 d specimen produced with 19 mm fiber at 0.3% content was 0.075 MPa. When the fiber content was 0.3%, the maximum indirect tensile strength of the 28 d solidified soil was 0.189 MPa.

Justifications of the results

Because fibers are stable materials, they do not react with silt or calcium carbide slag, and their effect on soil is similar to the mechanism by which plant roots solidify in soil. According to the bending mechanism, the bending fibers between soil particles limit the change in the relative position of the soil particles through the pressure and frictional force on the concave side of the soil particles, stabilizing the soil. According to the intercrossing mechanism, the intercrossing fibers in soil form a spatial network structure, and the action of external forces in one direction is counteracted by the entire network structure. In addition, the incorporation of fibers effectively reduces the internal pores of solidified soil, densifies the soil, and improves its mechanical properties; this can be called the “filling mechanism.” Based on the bending, intercrossing, and filling mechanisms, the preconditions for fiber stability in the soil are that the fiber must have a sufficient length and minimum distribution density in the soil.

Discussion

Effect of fiber on unconfined compressive strength of solidified soil

Based on the stabilization mechanism of fibers on soil, fibers do not affect the cementing action between the carbide slag and soil. Additionally, the unconfined compressive strength of carbide-slag-solidified soil is mainly influenced by the degree of soil compaction, carbide slag dosage, and soil group [9]. Hence, the fiber had no significant effect on the unconfined compressive strength of the solidified soil. The maximum unconfined compressive strength of the 7 d cured soils were observed when 19 mm fiber was added at a 0.4% content. Compared with the cured soil without fiber, the unconfined compressive strength increased by 8.16%. The fibers filled the pores between the particles of the solidified soil mixture and improved the density of the solidified soil, and reduced the void ratio of the solidified soil. Under the action of external load, the contact area between the fibers and the particles of the solidified soil mixture would generate friction, which played a constraint role on the slip of the particles of the solidified soil mixture. Therefore, the unconfined compressive strength of the 7 d solidified soil reinforced with fibers increased. Its increasing value was related to fiber incorporation: When the content of fiber was higher, the contact area between the fibers and the particles of the solidified soil mixture was greater, the friction would be more evident, and the enhancement of the unconfined compressive strength would be more evident. The maximum unconfined compressive strength of the 28 d solidified soil was observed when a 0.4% content of the 19 mm fiber was added. Compared with the solidified soil without fiber, the unconfined compressive strength increased by 13.16%. With increasing curing time, cementation reaction between the carbide slag and soil particles occurred, and the newly generated reaction products adhered to the spatial network structure formed by the fibers, limiting soil deformation and increasing the unconfined compressive strength of the soil. For the same carbide slag content and soil quality, the intercrossing mechanism effect became more significant with

increasing fiber content and length. Moreover, the spatial network structure stabilized further, and the increase in the unconfined compressive strength of the solidified soil became significant. However, when the fiber content exceeded a specific value, the fibers were less uniformly distributed in the soil, and the spatial network structure became less stable.

Effect of fiber on indirect tensile strength of solidified soil

Based on the stability mechanism of the fiber to the soil, the fiber did not affect the cementation between the carbide slag and soil. The effect of the fiber on the indirect tensile strength of the carbide-slag-solidified soil was mainly achieved by bending and intercrossing. For the 7 d solidified soil, the cementing action between the carbide slag and soil particles was incomplete, and an effective bond between the fiber and solidified soil was not established. Therefore, the effect of the fiber on the indirect tensile strength of the 7 d solidified soil was insignificant. The maximum indirect tensile strength of the 7 d solidified soil was observed for the fiber length of 19 mm (Fig 9). When the added amount was 0.3%, the indirect tensile strength of the solidified soil increased by 15.38% compared with that of the solidified soil without fiber. However, when the fiber content was 0.4%, the indirect tensile strength of the solidified soil decreased. This trend occurred because when the fiber length was extended, a high quantity of the fiber in the stabilized soil was difficult to distribute uniformly, easily formed clusters, reduced the bending effect, and intertwined. For the 28 d solidified soil, cementing between the carbide slag and soil particles was relatively complete, and adequate bonding between the fiber and solidified soil was achieved. Therefore, the fiber significantly increased the indirect tensile strength of the 28 d solidified soil. The maximum indirect tensile strength of the 28 d solidified soil was observed using 19 mm long fibers. When the added

amount was 0.3%, the indirect tensile strength of the solidified soil was 76.64% higher than that of the solidified soil without fibers. However, when the fiber content was increased to 0.4%, the indirect tensile strength of the solidified soil decreased, mainly because of difficulty in achieving uniform distribution with a relatively high amount of extended fibers; this easily induced the cluster phenomenon, reduced the bending effect, and caused intertwining.

Effect of fiber distribution density on mechanical properties of solidified soil

In this study, three fiber lengths (6, 12, and 19 mm) were adopted, and the incorporation amounts were 0.1%, 0.2%, 0.3%, and 0.4% of the total mass of solidified soil. The mass density of fibers in the solidified soil is denoted σ_{f} , and the fibers of lengths 6, 12, 19 mm are denoted σ_{f6} , σ_{f12} , and σ_{f19} , respectively. The number of fibers in the solidified soil per unit volume is denoted N_{f} , and the number of fibers of lengths 6, 12, and 19 mm are denoted N_{f6}, N_{f12}, and N_{f19}, respectively. In this study, the diameters for the three fiber lengths (6, 12, and 19 mm) were consistent when the σ_{f} of the specimens were the same: $N_{f6} = 2N_{f12} = 3N_{f19}$; that is, for an equal mass, the shorter the fiber, the more the number of fibers in the unit volume of the solidified soil. When the N_{f} values of the specimens were the same, $\sigma_{f19} = 1.5\sigma_{f12} = 3\sigma_{f6}$; that is, in the case of an equal number of fibers in the unit-volume solidified soil, the longer the fiber, the greater the mass density of fiber in the solidified soil of unit volume.

When the mass density of the 6 mm fiber in the solidified soil was $\sigma_{f6} = 0.1\%$, the mass density of fiber in the solidified soil reinforced with 12 and 19 mm fibers were $\sigma_{f12} = 0.2\%$ and

$\sigma_{f19} = 0.3\%$, respectively. The results of the unconfined compressive test obtained at 7 and 28 d are presented in Fig 10, and those of the indirect tensile test are presented in Fig 11.

Under the same conditions, the unconfined compressive strength of the 7 d solidified soil slightly increased with the fiber content (Fig 10). The unconfined compressive strength of the solidified soil reinforced with 19 mm fibers was the highest but only 7.14% higher than that without fibers. The main reason for this was that for the short curing period, the fibers did not form an effective bond with the solidified soil, and the bending and intercrossing actions were somewhat challenging to achieve. The increased unconfined compressive strength was mainly attributed to the effective reduction of the pores in the solidified soil by the incorporated fibers and the resulting filling effect, densifying the solidified soil. The solidified soil reinforced with 19 mm fibers using $\sigma_{f19} = 0.3\%$ had the highest unconfined compressive strength. The unconfined compressive strength of each solidified soil increased with the fiber content. The unconfined compressive strength of the solidified soil reinforced with 19 mm long fibers was the largest and was 12.5% higher than that of the solidified soil without fibers. This behavior was mainly attributed to the adequate bonding between the fiber and 28 d solidified soil and reflected the bending and intercrossing actions. The 19 mm fibers in the solidified soil were the longest, and the content, $\sigma_{f19} = 0.3\%$, was the largest. Moreover, the unconfined compressive strength was the highest under the combined actions of filling, bending, and intercrossing.

Under the same N_f conditions, the indirect tensile strength of the 7 d solidified soil increased with the fiber content (Fig 11). The indirect tensile strength of the solidified soil containing 19 mm long fibers was the highest; this strength was 15.38% higher than that of the solidified soil without fibers. The 28 d indirect tensile strength of each solidified soil increased

significantly with the incorporation of fibers. The solidified soil containing 19 mm long fiber exhibited the highest indirect tensile strength, which was 24.34% higher than that of the solidified soil without fibers. The results indicated that the fiber and solidified soil formed an effective bond with extended curing time, and the bending and intercrossing actions were significant.

A comparison between Figs 10 and 11 revealed that the increase in the indirect tensile strength of the fiber-reinforced solidified soil was significantly higher than that in the unconfined compression strength.

Conclusions

In this study, the effects of fiber length and fiber content on the properties of carbide-slag-solidified soil was investigated based on unconfined compressive strength and indirect tensile strength tests. The following conclusions were drawn.

(1) The fibers did not significantly increase the unconfined compressive and indirect tensile strengths of the carbide-slag-solidified soil at 7 d but effectively increased those at 28 d. When 19 mm long fibers were incorporated into the carbide-slag-solidified soil, the effect became most significant for the fiber content of 0.3%.

(2) The increase in the indirect tensile strength of the carbide-slag-solidified soil by the fibers was significantly higher than that in the unconfined compressive strength.

(3) For short-period (7 d) curing, the stabilization of the carbide-slag-solidified soil by the fibers mainly depended on the filling action. For long-age (28 d) curing, the stabilization by the fibers was caused by the combined actions of filling, intercrossing and bending.

References

1. Horpibulsuk S, Kampala A, Phetchuay C, Udomchai A, Arulrajah A. Calcium carbide residue-A cementing agent for sustainable soil stabilization. Geotech Eng J SEAGS AGSSEA. 2015;46: 22-27.
2. Liu YY, Chang CW, Namdar A, She Y, Lin C, Yuan X, et al. Stabilization of expansive soil using cementing material from rice husk ash and calcium carbide residue. Constr Build Mater. 2019;221: 1-11. doi: 10.1016/j.conbuildmat.2019.05.157.
3. Latifi N, Vahedifard F, Ghazanfari E, A. Rashid AS. Sustainable usage of calcium carbide residue for stabilization of clays. J Mater Civ Eng. 2018;30: 04018099. doi: 10.1061/(ASCE)MT.1943-5533.0002313.
4. Kampala A, Horpibulsuk S, Chinkullijniwat A, Shen S. Engineering properties of recycled calcium carbide Residue stabilized clay as fill and pavement materials. Constr Build Mater. 2013;46: 203-210. doi: 10.1016/j.conbuildmat.2013.04.037.
5. Kampala A, Horpibulsuk S. Engineering properties of silty clay stabilized with calcium carbide residue. J Mater Civ Eng. 2013;25: 632-644. Available from: https://doi/abs/10.1061/(ASCE)MT.1943-5533.0000618. doi: 10.1061/(ASCE)MT.1943-5533.0000618.
6. Kampala A, Horpibulsuk S, Prongmanee N, Chinkulkijniwat A. Influence of wet-dry cycles on compressive strength of calcium carbide residue-fly ash stabilized clay. J Mater Civ Eng. 2014;26: 633-643. Available from: https://org/doi/abs/10.1061/(ASCE)MT.1943-5533.0000853. doi: 10.1061/(ASCE)MT.1943-5533.0000853.
7. Li GX, Chen L, Zheng JQ, Jie YX. Experimental study on fiber reinforced cohesive soil. J

Hydraul Eng. 1995;06: 31-36. Available from: https://org/doi/10.3321/j.issn:0559-9350.1995.06.005.

8. Guo SA. Experimental study on shear strength of glass fiber reinforced lime soil. Highway Eng. 2019;44: 207-209 + 232. doi: 10.19782/j.cnki.1674-0610.2019.05.039.

9. Viswanadham BVS, Jha BK, Pawar SN. Influence of geofibers on the flexural behavior of compacted soil beams. Geosynthetics Int. 2010;17: 86-99. doi: 10.1680/gein.2010.17.2.86.

10. Kar R, Pradhan P. Strength and compressibility characteristics of randomly distributed fiber-reinforced soil. Int J Geotech Eng. 2011;5: 235-243. doi: 10.3328/IJGE.2011.05.02.235-243.

11. Cheng QQ, Zhang JX, Zhou N, Guo Y, Pan S. Experimental study on unconfined compression strength of polypropylene fiber reinforced composite cemented clay. Crystals. 2020;10: 247. doi: 10.3390/cryst10040247.

12. Li LH, Zang TB, Xiao HL, Feng W, Liu Y. Experimental study of polypropylene fiber reinforced clay soil mixed with municipal solid waste incineration bottom ash. Eur J Environ Civ Eng. 2020: 1-17. doi: 10.1080/19648189.2020.1795726.

13. Taha MMM, Feng CP, Ahmed SHS. Influence of polypropylene fiber (PF) reinforcement on mechanical properties of clay soil. Adv Polym Technol. 2020;2020: 1-15. doi: 10.1155/2020/9512839.

14. Li PL, Zhao CX, Pei Y, Hu JC. Composition design and influence factors of calcium carbide slag stabilized soil. Highway Eng. 2021;46: 129-133 + 255. doi: 10.19782/j.cnki.1674-0610.2021.03.020.

15. Tang CS, Shi B, Gu K. Microstructural study on interfacial interactions between fiber reinforcement and soil. J Eng Geol. 2011;19: 610-614. doi: 10.3969/j.issn.1004-9665.2011.04.026.

16. Zhang YM, Zhang XD, Zhang HR. Test research of geotechnique textile soil reinforcement mechanism and engineering application. Rock Soil Mech. 2005;08: 1323-1326. doi: 10.16285/j.rsm.2005.08.029.

17. Gong LX, Nie L, Xu Y. Discrete element analysis of the strength anisotropy of fiber - reinforced sands subjected to direct shear load. Appl Sci. 2020;10: 3693. doi: 10.3390/app10113693.

18. Beriha B, Sahoo UC. Analysis of flexible pavement with cross-anisotropic material properties. Int J Pavement Res Technol. 2020;13: 411-416. doi: 10.1007/s42947-020-0284-9.

19. Sujatha ER, Atchaya P, Darshan S, Subhashini S. Mechanical properties of glass fiber reinforced soil and its application as subgrade reinforcement. Road Mater Pavement Des. 2021;22: 2384–2395. doi: 10.1080/14680629.2020.1746387.

20. JTG 3430-2020. Test Methods of soils for Highway Engineering, Ministry of Transport of the People's Republic of China , Beijing, China. Available online: https://xxgk.mot.gov.cn/2020/jigou/glj/202008/t20200818_3452949.html (accessed on 10 August 2017).

21. GB/T 14336-2008. Testing Method for Length of Man-made Staple Fibers, General Administration of Quality Supervision, Inspection and Quarantine of the People's Republic of China, Beijing, China. Available online: http://www.csres.com/detail/192443.html (accessed on 18 June 2008).

22. GB/T 14337-2008. Testing Method for Length of Man-made Staple Fibers, Testing Method for Tensile Properties of Man-made Staple Fibers, Inspection and Quarantine of the People's Republic of China, Beijing, China. Available online: http://www.csres.com/detail/192444.html (accessed on 18 June 2008).

Funding: This study was funded by the Science and Technology Support Project of Langfang (Grant Number 2021013167).

Author contributions

Conceptualization: Hongzhou Zhang.

Data curation: Limei Tian, Shuang Wang, Yanhong Qiao.

Formal analysis: Limei Tian, Shuang Wang.

Writing – original draft: Hongzhou Zhang.

Writing – review & editing: Hongzhou Zhang.

Competing interests

The authors declare that no competing interests exist.

Figure

Click here to access/download;Figure;Figures.docx

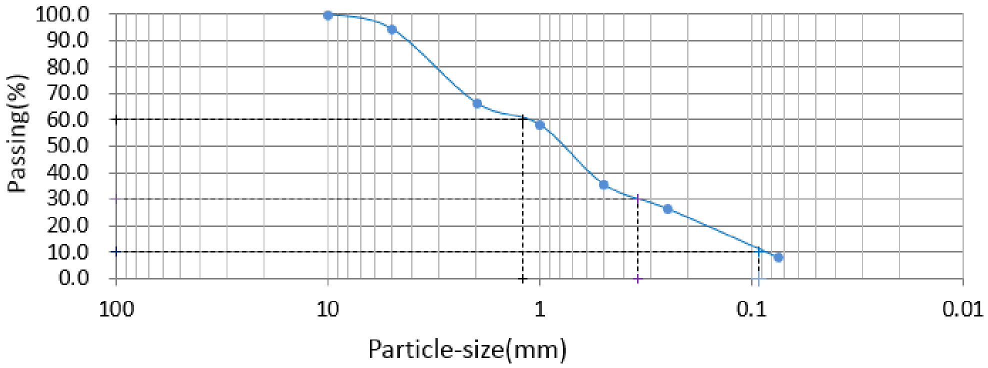

Fig 1. Particle-size distribution the soil.

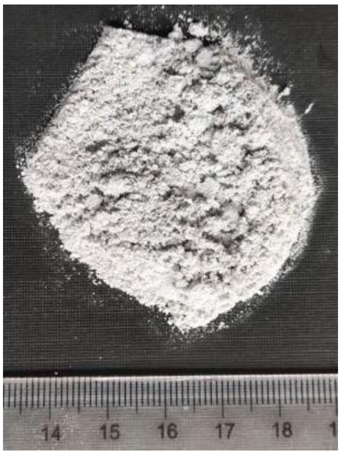

Fig 2. The carbide slag used in the study.

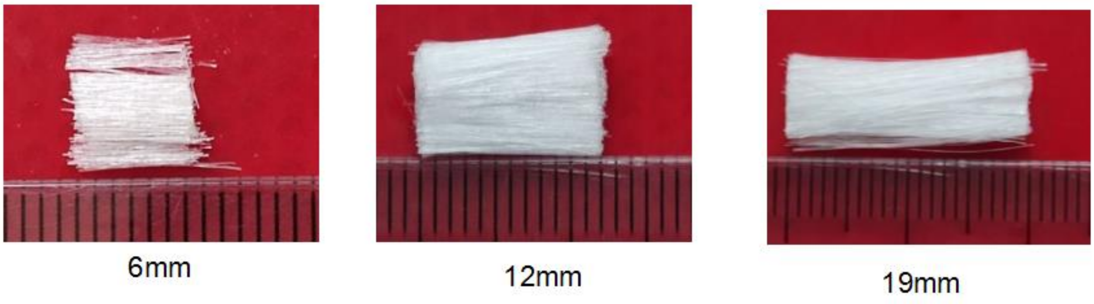

Fig 3. The fibers used for the study.

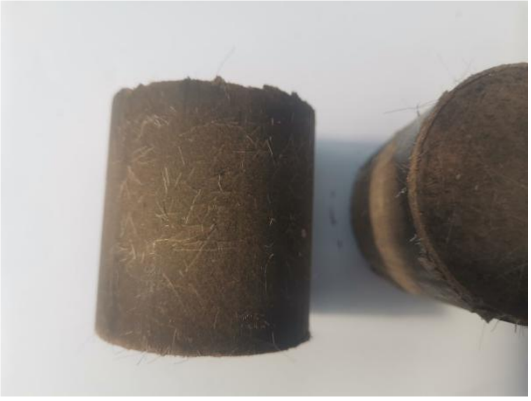

Fig 4. Prepared specimen of fiber stabilized carbide slag solidified soil.

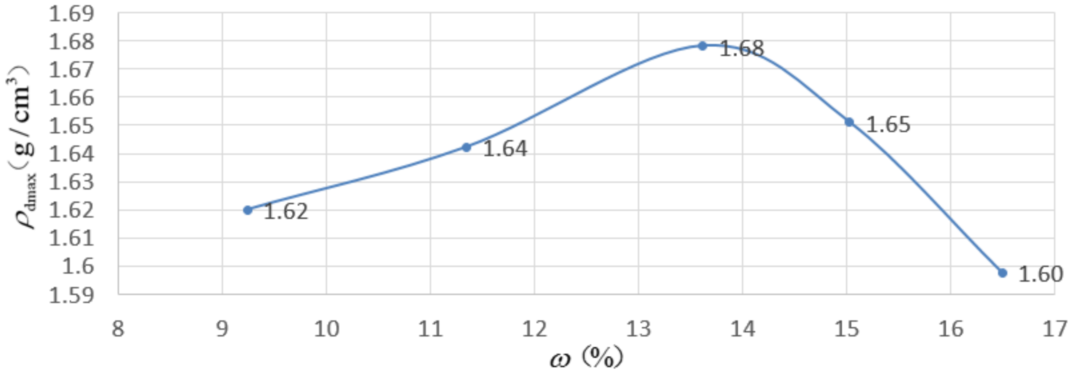

Fig 5. Compaction Curve of Carbide Slag Solidified Soil.

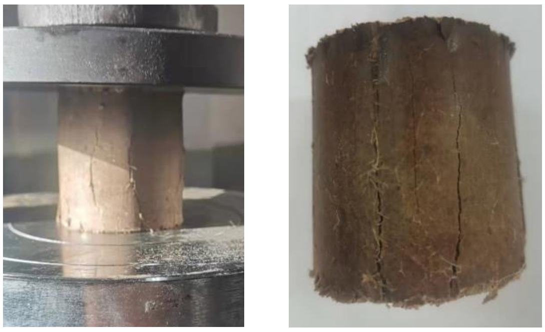

Fig 6. Failure of the Unconfined Compressive Test specimen.

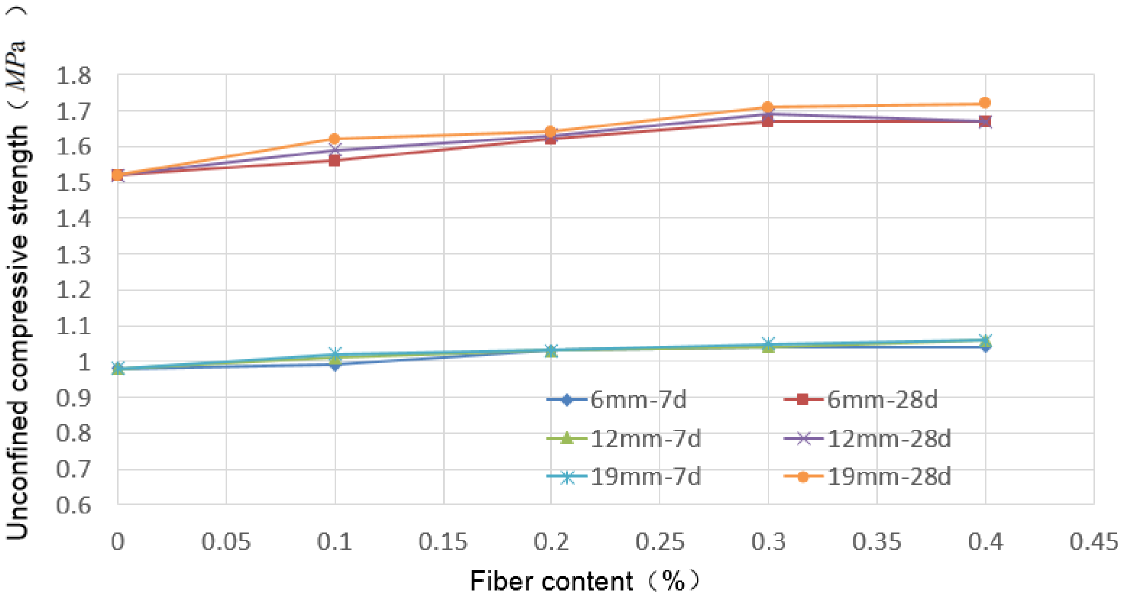

Fig 7. Unconfined Compressive Strength of fiber stabilized carbide slag solidified soil.

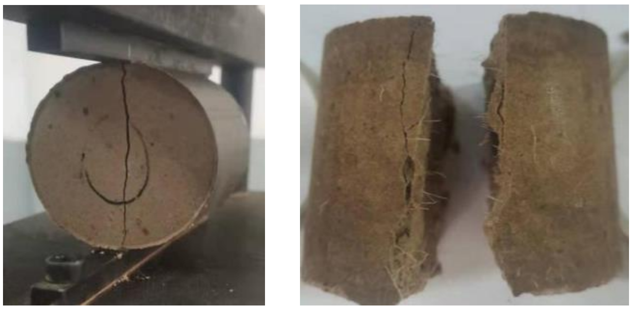

Fig.8. Indirect Tensile Strength test specimen.

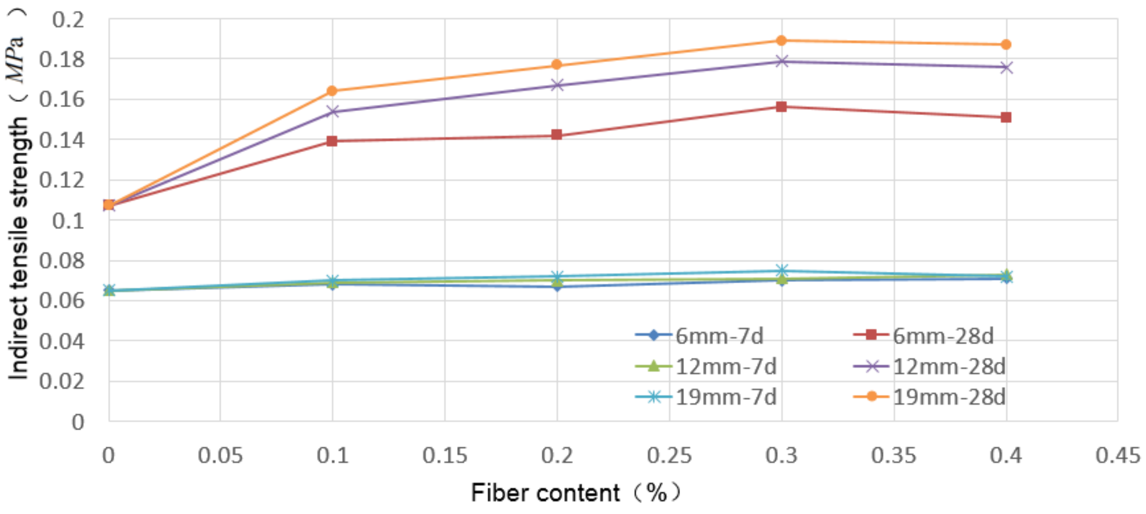

Fig 9. Results of Indirect Tensile Strength Test.

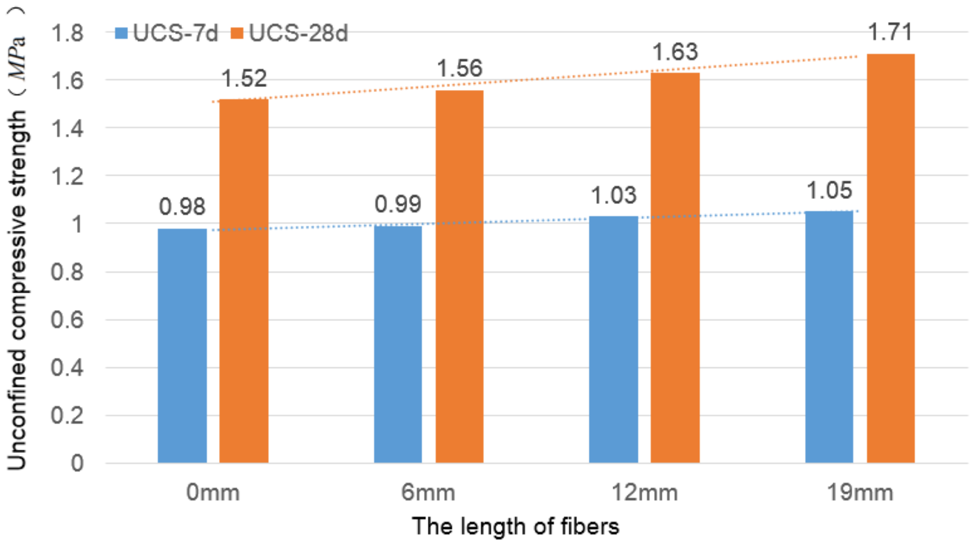

Fig 10. Results of Unconfined Compressive Test with same N_f.

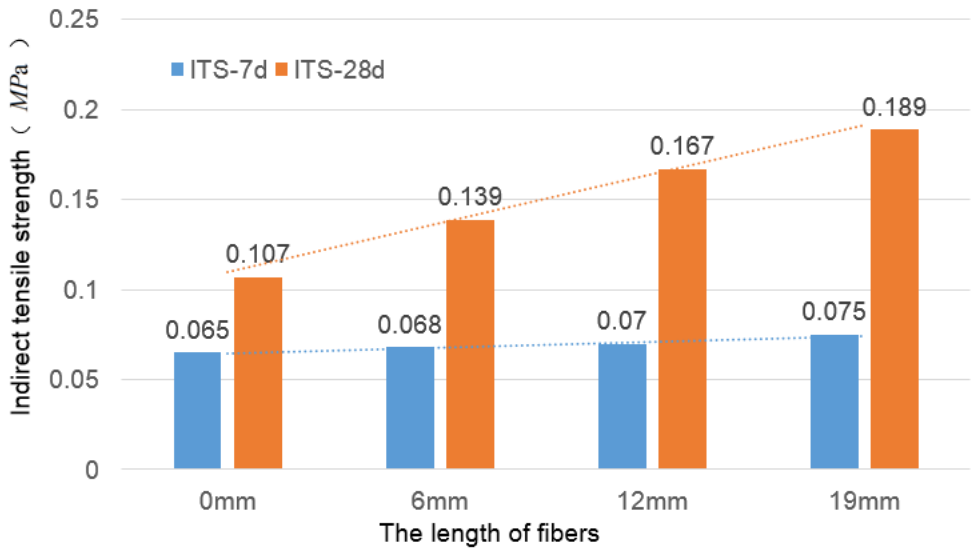

Fig 11. Results of Indirect Tensile Test with same N_f.

Click here to access/download
Supporting Information
supporting information.docx

Experimental study on engineering properties of fiber-stabilized carbide-slag-solidified soil

Hongzhou Zhang[1,2*], Limei Tian[2], Shuang Wang[2], Yanhong Qiao[2]

[1] China University of Geosciences Beijing, Beijing, China

[2] Langfang Normal University, Langfang, China

[*] Corresponding author

E-mail: zhanghongzhou@lfnu.edu.cn (HZ)

Abstract

Carbide slag has been used to prepare solidified soil to effectively reduce the stacking and disposal of carbide slag and achieve efficient resource utilization. Because of the significant brittleness and low strength of carbide-slag-stabilized soil, fibers were added to carbide-slag-stabilized soil in this experimental study. The effects of fiber length and fiber content on the unconfined compressive and indirect tensile strengths of carbide-slag-stabilized soil were investigated. The concepts of the density of fibers in solidified soil and the number of fibers in a unit volume solidified soil were proposed, and the effects of fiber distribution density on the mechanical properties of the solidified soil were evaluated. The fibers increased the indirect tensile strength of the carbide-slag-solidified soil, which was significantly higher than the unconfined compressive strength of the solidified soil. The fibers had no significant effect on the unconfined compressive and indirect tensile strengths of

Commented [zh1]: remove the mass

Commented [zh2]: remove the root

the 7 d carbide-slag-solidified soil but increased those of the 28 d carbide-slag-solidified soil. The enhancement effect was the most significant when a 0.3% content of 19 mm long fibers was incorporated into the carbide-slag-solidified soil.

Keywords

fiber; carbide slag; unconfined compressive strength; indirect tensile strength; solidified soil

Introduction

The efficient treatment and utilization of carbide slag is the only approach to solve the environmental pollution problem of the carbide industry and achieve sustainable development. Many researchers have attempted to utilize calcium carbide slag for soil reinforcement [1–3], and the solidified soil has problems, such as low strength and toughness and susceptibility to cracking [4]. After more than four wetting−drying cycles, the compressive strength of the carbide-slag-stabilized soil fails to satisfy the strength requirements of the subgrade [5,6].

Using fibers to stabilize soil is an effective soil reinforcement technique. Fibers can significantly increase the strength and toughness of soil and solve soil cracking problems [7,8]. The addition of fibers effectively improves the tensile strain and reduces the cracking of specimens [9]. Artificial and natural fibers significantly improve the residual shear strength and unconfined compressive strength limits of soil [10]. Polypropylene fibers can improve the compressive strength, shear strength, and deformation resistance of cement-clay [11–13]. The incorporation of fibers had obvious effect on delaying the cracking of carbide slag stabilized soil specimens under load. [14]. The reinforcement effect of the fiber depends on the strength of the interface between the reinforcement and soil, and the mechanical interactions between the reinforcement and soil are

Commented [zh3]: "Unconfined Compression Strength" change to " Unconfined Compressive Strength";

Commented [zh4]: "mass density of fiber in solidified soil" change to " solidified soil ";
"root number of fiber in unit volume solidified soil" had been removed.

Commented [zh5]:

Commented [zh6]: "improve the compaction strength" had been changed to " improve the compressive strength".

Commented [zh7]: "cemented clay" had been corrected to "cement-clay".

Commented [zh8]: " the load cracking " had been corrected to an appropriate expression.

mainly adhesion and friction [15]. The stability mechanism of fibers in the soil can be summarized as “bending mechanism” and “intercrossing mechanism” [16]. The reinforcement effect of fibers in the soil is anisotropic [17]. When the orientation angle of a fiber is perpendicular to the direction of the strong contact force, the fiber exerts the maximum effect [18]. Fibers can improve the ductility and compressive resistance of reinforced soil and reduce the sensitivity of soil to water [19]. Currently, research on the effects of fiber length and fiber content on the properties of fiber-stabilized carbide-slag-solidified soil has not been conducted extensively.

Commented [zh9]: " interlacing, interweaving, interleaving " were uniformly corrected as “intercrossing”.

In this study, fiber-stabilized carbide-slag-solidified silt was adopted as the research object. Based on unconfined compressive strength and indirect tensile strength tests, the effects of fiber length and fiber content on the performance of carbide-slag-solidified silt was investigated, and the optimum fiber length and content were determined. The concepts of the mass density of fibers in the solidified soil (DFS) and the root number of fibers in a unit volume solidified soil (NFS) were proposed. Moreover, the effects of fiber distribution density on the mechanical properties of the solidified soil were evaluated.

In this study, the properties of fiber-carbide slag-solidified silt were studied according to fiber length and fiber content. The research findings are expected to serve as a reference for applying fiber-stabilized carbide-slag-solidified soil in road engineering. It can protect the environment, improve the resource utilization efficiency of carbide slag, reduce the project cost, achieve sustainable development, and achieve remarkable economic and social benefits.

Commented [zh10]: Emphasize the novelty and importance of the work.

Materials and methods

Materials

The soil samples used for the tests were collected from the Airport, New Area of Langfang, Hebei Province, China. The study did not involve private or protected land. According to Test Methods of soils for Highway Engineering (JTG 3430-2020) [20], the physical indexes and particle gradation of test soil are determined. The physical indexes are listed in Table 1, and the particle size distribution is shown in Fig 1, where C_u and C_c are 13 and 1.06, respectively. Before the tests, the soil samples were crushed by soil milling and passed through a 2.36 mm square hole sieve. The soil collected under the sieve was used for testing.

Commented [zh11]: Standards used in these tests had been added and properly referenced.

Table 1. Physical Indexes of Soil Used for Testing.

Property	ω_L %	ω_P %	I_P %	G	σ_{dmax} g/cm^3	OMC %
**Value**	31.1	22.8	8.3	2.45	1.86	12.15

Commented [zh12]: The errors had been corrected。LL, PL…etc.

Carbide slag was purchased from Dezhou Shihua Chemical Co., Ltd. The raw calcium carbide slag had a high moisture content and a peculiar smell. After drying and dehydration, it was crushed by soil milling and passed through a 0.075 mm square hole sieve. The carbide slag under the sieve was used for the tests (Fig 2). The main components of the calcium carbide slag are listed in Table 2. The carbide slag contained a large amount of calcium oxide similar to that of quick lime and exhibited significant activity (Table 2).

Chemical compound	CaO	SiO_2	Al_2O_3	Fe_2O_3	MgO	Loss on ignition

**Content (%)**	65.77	3.15	1.78	0.24	0.14	23.56

Table 2. Chemical Composition of Carbide Slag.

The fibers were purchased from Changsha Ninxiang Building Materials Co., Ltd. Polypropylene fibers with lengths of 6, 12, and 19 mm were used for testing (Fig 3). According to Testing Method for Length of Man-made Staple Fibers(GB/T 14336-2008) [21] and Testing Method for Tensile Properties of Man-made Staple Fibers(GB/T 14337-2008) [22]，the physical and mechanical properties of the fibers are listed in Table 3.

Commented [zh13]: Standards used in these tests had been added and properly referenced.

Table 3. Physical and Mechanical Properties of Polypropylene Fibers.

**Length (mm)**	**Diameter (µm)**	**Density (g/cm^3)**	**Tensile strength (*MP*a)**	***E* (*MP*a)**	**Ultimate elongation (%)**	**Melting point (°C)**
6/12/19	32.7	0.91	469	4236	28.4	169

Methods

This study was divided into three steps to investigate the effects of the fiber length and content on the performance of fiber-stabilized carbide-slag-solidified silt, optimal fiber length and content, and effect of fiber distribution density on the mechanical properties of the solidified soil.

(1) Compaction tests. The optimum moisture content and the maximum dry density of carbide-slag-solidified silt were determined according to “Compaction Test Method for Inorganic Binder Stabilized Materials” (T0804-1994).

Commented [zh14]: It had been modified according to reviewer’s comment.

(2) Unconfined compressive strength tests. The unconfined compressive strength of fiber-stabilized carbide-slag-solidified silt was determined according to the "Unconfined Compressive Strength Test Method for Inorganic Binder Stabilized Materials" (T0805-1994).

(3) Indirect tensile strength test. The indirect tensile strength of fiber-stabilized calcium-carbide-slag-solidified silt was determined according to "Indirect Tensile Strength Test Method for Inorganic Binder Stabilized Materials (Splitting Test)" (JTGE51-2009-T0806).

Preparation of specimens

The specimens were prepared using the method specified in "Inorganic Binder-Stabilized Material Specimen Production Method (Cylindrical)" (T0843-2009). Dried sieved carbide slag and silt were thoroughly blended at a mass ratio of 2:8. An appropriate amount of the fiber was proportionally added to the mixture and thoroughly stirred manually to fully disperse the fiber into the mixture. An appropriate amount of water was added to the mixture blended with the fiber, stirred evenly, and placed in a plastic bag. The bag was sealed and placed in the dark for 24 h. A 50 mm × 50 mm test mold was adopted, and the calculated weight of the soil sample was determined based on the degree of compaction (96%). Twelve samples were prepared for each group. After the specimens were produced using the static pressure method, they were placed in a curing box (temperature = 20±2 °C; relative humidity ≥ 95%). The specimens are shown in Fig 4. The proportions of the specimens of fiber-stabilized carbide-slag-solidified soil are listed in Table 4.

Table 4. Proportions of Specimens.

Fiber length (mm)	Fiber content (%)	Curing period (d)	Parameter
6	0/0.1/0.2/0.3/0.4	7/28	Unconfined compressive strength test<br>Indirect tensile strength test
12	0.1/0.2/0.3/0.4	7/28	Unconfined compressive strength test<br>Indirect tensile strength test
19	0.1/0.2/0.3/0.4	7/28	Unconfined compressive strength test<br>Indirect tensile strength test

Results

Compaction

According to the experiment, the optimum moisture content of 2:8 carbide-slag-solidified soil was 13%-14%. First, five portions of 2:8 carbide-slag-solidified soil were prepared, and the target moisture contents were 10%, 12%, 14%, 16%, and 18%. Compaction tests were performed according to the compaction test method for inorganic binder stabilization materials (T0804-1994). The compaction curve of the soil samples is shown in (Fig 5). The optimum moisture content of 2:8 carbide-slag-solidified soil was 13.62 %, and the maximum dry density was 1.68g/cm^3. Because the amount of fiber was small, its effects on the optimum moisture content and maximum dry density were not evident. Therefore, when preparing an unconfined compressive strength test and indirect tensile strength test samples, it could be directly configured according to the optimum water content of 13.62 % and the maximum dry density of 1.68g/cm^3.

Commented [zh15]: " experience" had been changed to "experiment"

Commented [zh16]: All "optimal moisture content" had been changed to "optimum moisture content".

Commented [zh17]: “the” change to “its”.

Commented [zh18]: “making” change to “preparing”

Unconfined compressive strength

The failure pattern of the specimens subjected to unconfined compression is shown in Fig 6. The unconfined compressive strength test results of 2:8 carbide-slag-solidified soil with different fiber lengths and contents at 7 and 28 d are shown in Fig 7.

The incorporation of fibers somewhat improved the unconfined compressive strength of the solidified soil, but the improvement was limited. For the same fiber length and content, the unconfined compressive strength of the solidified soil significantly increased with increasing curing time. For each group of fibers, the 7 and 28 d unconfined compressive strength increased with increasing fiber content, but the degree of improvement was limited. When the fiber contents were 0.3% and 0.4%, the variation in the unconfined compressive strength was insignificant. For the solidified soil reinforced with a 6 mm fiber at a 0.3% fiber content, the maximum values of the 7 and 28 d unconfined compressive strengths were 1.04 and 1.67 MPa, respectively. For the solidified soil mixed with a 12 mm fiber, the maximum value of the 7 d unconfined compressive strength (1.06 MPa) was observed at the 0.4% fiber content. The maximum unconfined compressive strength at 28 d was 1.69 MPa when the fiber content was 0.3%. For the solidified soil mixed with a 19 mm fiber, the maximum unconfined compressive strengths at 7 and 28 d were 1.06 and 1.72 MPa, respectively, when the fiber content was 0.4%. With an increase in the fiber length, the unconfined compressive strength of the solidified soil increased slightly, but the degree of increase was minimal.

Commented [zh19]: All “age” change to “curing time”.

Indirect tensile strength

The failure patterns of the test specimens subjected to indirect tensile strength test is shown in Fig 8. The indirect tensile strength test results for the calcium-carbide-slag-solidified soil at 7 and 28 d in 2:8 ratio with different fiber lengths and contents are presented in Fig 9. The addition of fiber did not influence the indirect tensile strength of the solidified soil at 7 d but significantly increased the indirect tensile strength at 28 d (Fig 9). For the same fiber length and content, the indirect tensile strength of the solidified soil significantly increased with increasing curing time. With an increase in the fiber length for the same fiber content, the indirect tensile strength of the 7 d solidified soil did not increase significantly; in contrast, the indirect tensile strength of the 28 d solidified soil increased significantly. When the fiber length was 6 mm, and the fiber content was 0.4%, the maximum indirect tensile strength at 7 d was 0.071 MPa. The maximum indirect tensile strength at 28 d was 0.156 MPa when the fiber content was 0.3%. When the fiber length was 12 mm using a fiber content of 0.3%, the maximum indirect tensile strength at 28 d was 0.156 MPa. The maximum indirect tensile strength of the 7 d cured soil was 0.073 MPa when the fiber content was 0.4%. The maximum indirect tensile strength of the cured soil at 28 d was 0.179 MPa when the fiber content was 0.3%. The maximum indirect tensile strength of the 7 d specimen produced with 19 mm fiber at 0.3% content was 0.075 MPa. When the fiber content was 0.3%, the maximum indirect tensile strength of the 28 d solidified soil was 0.189 MPa.

Justifications of the results

Because fibers are stable materials, they do not react with silt or calcium carbide slag, and their effect on soil is similar to the mechanism by which plant roots solidify in soil. According to the bending mechanism, the bending fibers between soil particles limit the change in the relative position of the soil particles through the pressure and frictional force on the concave side of the soil

particles, stabilizing the soil. According to the intercrossing mechanism, the intercrossing fibers in soil form a spatial network structure, and the action of external forces in one direction is counteracted by the entire network structure. In addition, the incorporation of fibers effectively reduces the internal pores of solidified soil, densifies the soil, and improves its mechanical properties; this can be called the “filling mechanism.” Based on the bending, intercrossing, and filling mechanisms, the preconditions for fiber stability in the soil are that the fiber must have a sufficient length and minimum distribution density in the soil.

Discussion

Effect of fiber on unconfined compressive strength of solidified soil

Based on the stabilization mechanism of fibers on soil, fibers do not affect the cementing action between the carbide slag and soil. Additionally, the unconfined compressive strength of carbide-slag-solidified soil is mainly influenced by the degree of soil compaction, carbide slag dosage, and soil group [9]. Hence, the fiber had no significant effect on the unconfined compressive strength of the solidified soil. The maximum unconfined compressive strength of the 7 d cured soils were observed when 19 mm fiber was added at a 0.4% content. Compared with the cured soil without fiber, the unconfined compressive strength increased by 8.16%. The fibers filled the pores between the particles of the solidified soil mixture and improved the density of the solidified soil, and reduced the void ratio of the solidified soil. Under the action of external load, the contact area between the fibers and the particles of the solidified soil mixture would generate friction, which played a constraint role on the slip of the particles of the solidified soil mixture. Therefore, the unconfined compressive strength of the 7 d solidified soil reinforced with fibers increased. Its

Commented [zh20]: "compacts the soil" change to " densifiesthe soil "

Commented [zh21]: “compacts” change to “densifies”

Commented [zh22]: Add **Justifications** of the results

Commented [zh23]: ” Gelatin” had been changed to “Cementing Action”.

Commented [zh24]:

Commented [zh25]: “soil quality” had been changed to “soil group”.

Commented [zh26]: “compactness” had been changed to “density”.

increasing value was related to fiber incorporation: When the content of fiber was higher, the contact area between the fibers and the particles of the solidified soil mixture was greater, the friction would be more evident, and the enhancement of the unconfined compressive strength would be more evident. The maximum unconfined compressive strength of the 28 d solidified soil was observed when a 0.4% content of the 19 mm fiber was added. Compared with the solidified soil without fiber, the unconfined compressive strength increased by 13.16%. With increasing curing time, cementation reaction between the carbide slag and soil particles occurred, and the newly generated reaction products adhered to the spatial network structure formed by the fibers, limiting soil deformation and increasing the unconfined compressive strength of the soil. For the same carbide slag content and soil quality, the intercrossing mechanism effect became more significant with increasing fiber content and length. Moreover, the spatial network structure stabilized further, and the increase in the unconfined compressive strength of the solidified soil became significant. However, when the fiber content exceeded a specific value, the fibers were less uniformly distributed in the soil, and the spatial network structure became less stable.

Commented [zh27]: We had re-written this part according to the Reviewer's suggestion.

Commented [zh28]:

Commented [zh29]: "aging" changed to "increasing curing time"

Effect of fiber on indirect tensile strength of solidified soil

Based on the stability mechanism of the fiber to the soil, the fiber did not affect the cementation between the carbide slag and soil. The effect of the fiber on the indirect tensile strength of the carbide-slag-solidified soil was mainly achieved by bending and intercrossing. For the 7 d solidified soil, the cementing action between the carbide slag and soil particles was incomplete, and an effective bond between the fiber and solidified soil was not established. Therefore, the effect of the fiber on the indirect tensile strength of the 7 d solidified soil was insignificant. The maximum

indirect tensile strength of the 7 d solidified soil was observed for the fiber length of 19 mm (Fig 9). When the added amount was 0.3%, the indirect tensile strength of the solidified soil increased by 15.38% compared with that of the solidified soil without fiber. However, when the fiber content was 0.4%, the indirect tensile strength of the solidified soil decreased. This trend occurred because when the fiber length was extended, a high quantity of the fiber in the stabilized soil was difficult to distribute uniformly, easily formed clusters, reduced the bending effect, and intertwined. For the 28 d solidified soil, cementing between the carbide slag and soil particles was relatively complete, and adequate bonding between the fiber and solidified soil was achieved. Therefore, the fiber significantly increased the indirect tensile strength of the 28 d solidified soil. The maximum indirect tensile strength of the 28 d solidified soil was observed using 19 mm long fibers. When the added amount was 0.3%, the indirect tensile strength of the solidified soil was 76.64% higher than that of the solidified soil without fibers. However, when the fiber content was increased to 0.4%, the indirect tensile strength of the solidified soil decreased, mainly because of difficulty in achieving uniform distribution with a relatively high amount of extended fibers; this easily induced the cluster phenomenon, reduced the bending effect, and caused intertwining.

Effect of fiber distribution density on mechanical properties of solidified soil

In this study, three fiber lengths (6, 12, and 19 mm) were adopted, and the incorporation amounts were 0.1%, 0.2%, 0.3%, and 0.4% of the total mass of solidified soil. The mass density of fibers in the solidified soil is denoted σ_{f}, and the fibers of lengths 6, 12, 19 mm are denoted $\sigma_{\mathrm{f}6}$, $\sigma_{\mathrm{f}12}$, and $\sigma_{\mathrm{f}19}$, respectively. The number of fibers in the solidified soil per unit volume is denoted

Commented [zh30]: “fiber roots” change to “fibers”.

N_{f}, and the number of fibers of lengths 6, 12, and 19 mm are denoted $N_{\mathrm{f}6}$, $N_{\mathrm{f}12}$, and $N_{\mathrm{f}19}$, respectively. In this study, the diameters for the three fiber lengths (6, 12, and 19 mm) were consistent when the σ_{f} of the specimens were the same: $N_{\mathrm{f}6} = 2N_{\mathrm{f}12} = 3N_{\mathrm{f}19}$; that is, for an equal mass, the shorter the fiber, the more the number of fibers in the unit volume of the solidified soil. When the N_{f} values of the specimens were the same, $\sigma_{\mathrm{f}19} = 1.5\sigma_{\mathrm{f}12} = 3\sigma_{\mathrm{f}6}$; that is, in the case of an equal number of fibers in the unit-volume solidified soil, the longer the fiber, the greater the mass density of fiber in the solidified soil of unit volume.

When the mass density of the 6 mm fiber in the solidified soil was $\sigma_{\mathrm{f6}} = 0.1\%$, the mass density of fiber in the solidified soil reinforced with 12 and 19 mm fibers were $\sigma_{\mathrm{f12}} = 0.2\%$ and $\sigma_{\mathrm{f19}} = 0.3\%$, respectively. The results of the unconfined compressive test obtained at 7 and 28 d are presented in Fig 10, and those of the indirect tensile test are presented in Fig 11.

Under the same conditions, the unconfined compressive strength of the 7 d solidified soil slightly increased with the fiber content (Fig 10). The unconfined compressive strength of the solidified soil reinforced with 19 mm fibers was the highest but only 7.14% higher than that without fibers. The main reason for this was that for the short curing period, the fibers did not form an effective bond with the solidified soil, and the bending and intercrossing actions were somewhat challenging to achieve. The increased unconfined compressive strength was mainly attributed to the effective reduction of the pores in the solidified soil by the incorporated fibers and the resulting filling effect, densifying the solidified soil. The solidified soil reinforced with 19 mm fibers using $\sigma_{\mathrm{f19}} = 0.3\%$ had the highest unconfined compressive strength. The unconfined compressive strength of each solidified soil increased with the fiber content. The unconfined compressive strength of the solidified soil reinforced with 19 mm long fibers was the largest and was 12.5%

higher than that of the solidified soil without fibers. This behavior was mainly attributed to the adequate bonding between the fiber and 28 d solidified soil and reflected the bending and intercrossing actions. The 19 mm fibers in the solidified soil were the longest, and the content, $\sigma_{\mathrm{f19}} = 0.3\%$, was the largest. Moreover, the unconfined compressive strength was the highest under the combined actions of filling, bending, and intercrossing.

Commented [zh31]: All "interleaving effects" had been changed to "intercrossing actions".

Under the same N_{f} conditions, the indirect tensile strength of the 7 d solidified soil increased with the fiber content (Fig 11). The indirect tensile strength of the solidified soil containing 19 mm long fibers was the highest; this strength was 15.38% higher than that of the solidified soil without fibers. The 28 d indirect tensile strength of each solidified soil increased significantly with the incorporation of fibers. The solidified soil containing 19 mm long fiber exhibited the highest indirect tensile strength, which was 24.34% higher than that of the solidified soil without fibers. The results indicated that the fiber and solidified soil formed an effective bond with extended curing time, and the bending and intercrossing actions were significant.

A comparison between Figs 10 and 11 revealed that the increase in the indirect tensile strength of the fiber-reinforced solidified soil was significantly higher than that in the unconfined compression strength.

Commented [zh32]: " suggests that" change to " revealed that"

Conclusions

In this study, the effects of fiber length and fiber content on the properties of carbide-slag-solidified soil was investigated based on unconfined compressive strength and indirect tensile strength tests. The following conclusions were drawn.

(1) The fibers did not significantly increase the unconfined compressive and indirect tensile strengths of the carbide-slag-solidified soil at 7 d but effectively increased those at 28 d. When 19 mm long fibers were incorporated into the carbide-slag-solidified soil, the effect became most significant for the fiber content of 0.3%.

(2) The increase in the indirect tensile strength of the carbide-slag-solidified soil by the fibers was significantly higher than that in the unconfined compressive strength.

(3) For short-period (7 d) curing, the stabilization of the carbide-slag-solidified soil by the fibers mainly depended on the filling action. For long-age (28 d) curing, the stabilization by the fibers was caused by the combined actions of filling, intercrossing and bending.

References

1. Horpibulsuk S, Kampala A, Phetchuay C, Udomchai A, Arulrajah A. Calcium carbide residue-A cementing agent for sustainable soil stabilization. Geotech Eng J SEAGS AGSSEA. 2015;46: 22-27.

Commented [zh33]: give the full list of names

2. Liu YY, Chang CW, Namdar A, She Y, Lin C, Yuan X, et al. Stabilization of expansive soil using cementing material from rice husk ash and calcium carbide residue. Constr Build Mater. 2019;221: 1-11. doi: 10.1016/j.conbuildmat.2019.05.157.
3. Latifi N, Vahedifard F, Ghazanfari E, A. Rashid AS. Sustainable usage of calcium carbide residue for stabilization of clays. J Mater Civ Eng. 2018;30: 04018099. doi: 10.1061/(ASCE)MT.1943-5533.0002313.
4. Kampala A, Horpibulsuk S, Chinkullijniwat A, Shen S. Engineering properties of recycled calcium carbide Residue stabilized clay as fill and pavement materials. Constr Build Mater.

2013;46: 203-210. doi: 10.1016/j.conbuildmat.2013.04.037.

5. Kampala A, Horpibulsuk S. Engineering properties of silty clay stabilized with calcium carbide residue. J Mater Civ Eng. 2013;25: 632-644. Available from: https://doi/abs/10.1061/(ASCE)MT.1943-5533.0000618. doi: 10.1061/(ASCE)MT.1943-5533.0000618.

6. Kampala A, Horpibulsuk S, Prongmanee N, Chinkulkijniwat A. Influence of wet-dry cycles on compressive strength of calcium carbide residue-fly ash stabilized clay. J Mater Civ Eng. 2014;26: 633-643. Available from: https://org/doi/abs/10.1061/(ASCE)MT.1943-5533.0000853. doi: 10.1061/(ASCE)MT.1943-5533.0000853.

7. Li GX, Chen L, Zheng JQ, Jie YX. Experimental study on fiber reinforced cohesive soil. J Hydraul Eng. 1995;06: 31-36. Available from: https://org/doi/10.3321/j.issn:0559-9350.1995.06.005.

8. Guo SA. Experimental study on shear strength of glass fiber reinforced lime soil. Highway Eng. 2019;44: 207-209 + 232. doi: 10.19782/j.cnki.1674-0610.2019.05.039.

9. Viswanadham BVS, Jha BK, Pawar SN. Influence of geofibers on the flexural behavior of compacted soil beams. Geosynthetics Int. 2010;17: 86-99. doi: 10.1680/gein.2010.17.2.86.

10. Kar R, Pradhan P. Strength and compressibility characteristics of randomly distributed fiber-reinforced soil. Int J Geotech Eng. 2011;5: 235-243. doi: 10.3328/IJGE.2011.05.02.235-243.

11. Cheng QQ, Zhang JX, Zhou N, Guo Y, Pan S. Experimental study on unconfined compression strength of polypropylene fiber reinforced composite cemented clay. Crystals. 2020;10: 247. doi: 10.3390/cryst10040247.

12. Li LH, Zang TB, Xiao HL, Feng W, Liu Y. Experimental study of polypropylene fiber

reinforced clay soil mixed with municipal solid waste incineration bottom ash. Eur J Environ Civ Eng. 2020: 1-17. doi: 10.1080/19648189.2020.1795726.

13. Taha MMM, Feng CP, Ahmed SHS. Influence of polypropylene fiber (PF) reinforcement on mechanical properties of clay soil. Adv Polym Technol. 2020;2020: 1-15. doi: 10.1155/2020/9512839.

14. Li PL, Zhao CX, Pei Y, Hu JC. Composition design and influence factors of calcium carbide slag stabilized soil. Highway Eng. 2021;46: 129-133 + 255. doi: 10.19782/j.cnki.1674-0610.2021.03.020.

15. Tang CS, Shi B, Gu K. Microstructural study on interfacial interactions between fiber reinforcement and soil. J Eng Geol. 2011;19: 610-614. doi: 10.3969/j.issn.1004-9665.2011.04.026.

16. Zhang YM, Zhang XD, Zhang HR. Test research of geotechnique textile soil reinforcement mechanism and engineering application. Rock Soil Mech. 2005;08: 1323-1326. doi: 10.16285/j.rsm.2005.08.029.

17. Gong LX, Nie L, Xu Y. Discrete element analysis of the strength anisotropy of fiber - reinforced sands subjected to direct shear load. Appl Sci. 2020;10: 3693. doi: 10.3390/app10113693.

18. Beriha B, Sahoo UC. Analysis of flexible pavement with cross-anisotropic material properties. Int J Pavement Res Technol. 2020;13: 411-416. doi: 10.1007/s42947-020-0284-9.

19. Sujatha ER, Atchaya P, Darshan S, Subhashini S. Mechanical properties of glass fiber reinforced soil and its application as subgrade reinforcement. Road Mater Pavement Des. 2021;22: 2384–2395. doi: 10.1080/14680629.2020.1746387.

20. JTG 3430-2020. Test Methods of soils for Highway Engineering, Ministry of Transport of the People's Republic of China , Beijing, China. Available online: https://xxgk.mot.gov.cn/2020/jigou/glj/202008/t20200818_3452949.html (accessed on 10 August 2017).

21. GB/T 14336-2008. Testing Method for Length of Man-made Staple Fibers, General Administration of Quality Supervision, Inspection and Quarantine of the People's Republic of China, Beijing, China. Available online: http://www.csres.com/detail/192443.html (accessed on 18 June 2008).

22. GB/T 14337-2008. Testing Method for Length of Man-made Staple Fibers, Testing Method for Tensile Properties of Man-made Staple Fibers, Inspection and Quarantine of the People's Republic of China, Beijing, China. Available online: http://www.csres.com/detail/192444.html (accessed on 18 June 2008).

Funding: This study was funded by the Science and Technology Support Project of Langfang (Grant Number 2021013167), Science and Technology Support Project of Langfang (Grant Number 2021013162), and Science and Technology Support Project of Langfang Normal University (Grant Number XBQ202113).

Author contributions

Conceptualization: Hongzhou Zhang.

Data curation: Limei Tian, Shuang Wang, Yanhong Qiao.

Formal analysis: Limei Tian, Shuang Wang.

Writing – original draft: Hongzhou Zhang.

Writing – review & editing: Hongzhou Zhang.

Competing interests

The authors declare that no competing interests exist.

Dear Editor and Reviewers:

Thank you for your letter and for the reviewers' comments concerning our manuscript entitled "Experimental study on engineering properties of fiber-stabilized carbide-slag-solidified soil" (ID: PONE-D-22-00217). Those comments are all valuable and very helpful for revising and improving our paper, as well as the important guiding significance to our researches. We have studied comments carefully and have made correction which we hope meet with approval. Revised portion are marked in red in the paper. The main corrections in the paper and the responds to the reviewer's comments are as flowing:

Responds to the reviewer's comments:

Reviewer #1:

1. Response to comment: The novelty and importance of the work should be stressed.

Response: It had been modified according to reviewer's comment.

2. Response to comment: Table 1 and Fig. 1, standards used in these tests should be added and properly referenced.

Response: It had been modified according to reviewer's comment.

3. Response to comment: Table 1: there are errors in the titles LL, PL…etc.

Response: The errors had been corrected。

4. Response to comment: Standards used in testing of the fiber (Table 3) should be added.

Response: It had been modified according to reviewer's comment.

5. Response to comment: Quality of figures should be amended.

Response: It had been modified according to reviewer's comment.

6. Response to comment: Style of Figures 5, 7, 9, 10 and 11. The titles of the axes should be centered, legends of the figures should be enhanced, and numbers should be more visible.

Response: It had been modified according to reviewer's comment.

7. Response to comment: Justifications of the results should be added. The readers are more interested in the probable reasons of the expected behavior than only reporting the results.

Response: It had been modified according to reviewer’s comment.

Special thanks to you for your good comments.

Reviewer #2:

1. Response to comment: Page 1 line 19, in abstract "The concepts of mass density "the density is mass per unit volume the expression "mass density" is an incorrect expression remove the mass.

Response: It had been modified according to reviewer’s comment.

2. Response to comment: Page 1 line 19, in abstract "the root number of fibers " explain the root number? Is it just number of fibers?

Response: Yes, it is just number of fibers, the root had been removed.

3. Response to comment: All "Unconfined Compression Strength" change to 'unconfined compressive strength'

Response: It had been modified according to reviewer’s comment.

4. Response to comment: On Page #2 "Keywords: "Unconfined Compression Strength" change to " Unconfined Compressive Strength";

Response: It had been modified according to reviewer’s comment.

5. Response to comment: On Page #2 "Keywords: "mass density of fiber in solidified soil"; is a sentence not a keyword, change to " solidified soil ";

Response: It had been modified according to reviewer’s comment.

6. Response to comment: On Page #2 "Keywords: "root number of fiber in unit volume solidified soil", is a sentence not a keyword, it is suggested to be removed.

Response: It had been modified according to reviewer’s comment.

7. Response to comment: Page 3 line 50, "improve the compaction strength" change to " improve the compressive strength"

Response: It had been modified according to reviewer’s comment.

8. Response to comment: Page 3 line 51, " of cemented clay" is not clear is it clay stabilized with cement? Explain and correct it

Response: Yes, it is clay stabilized with cement, it had been corrected to "cement-clay".

9. Response to comment: Page 3 line 52, " the load cracking " is not an appropriate expression, correct it

Response: It had been modified according to reviewer’s comment.

10. Response to comment: Page 3 line 57, " interlacing mechanism " is not clear for me, explain that, several expressions used in the paper " interlacing, interweaving, interleaving, … what are the differences between them?

Response: There is no difference between " interlacing, interweaving, interleaving ", they were uniformly corrected as “intercrossing”.

Intercrossing mechanism refers to the fact that there are countless intercr ossing points of disordered fibers in the soil. If the fibers are subjected to force at the intersection of fibers, there is a trend of displacement, and ot her fibers will encounter to prevent this displacement. In other words, the stress and deformation of any segment of fiber will affect the fibers in all directions intertwined with it, thus forming the stress area of space. As sh own in Figure 1.

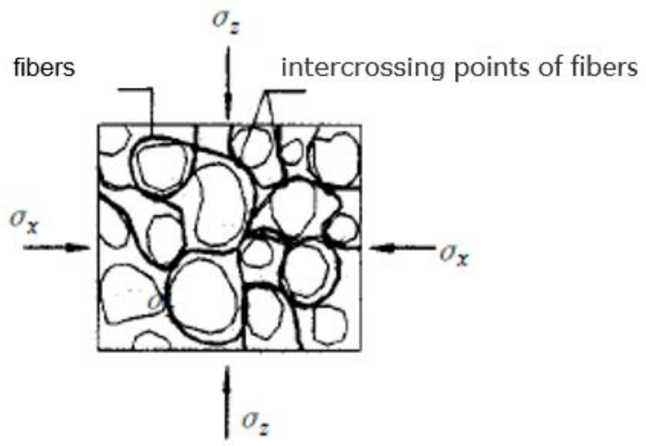

Figure 1 Intercrossing mechanism

11. Response to comment: Page 6 line 111, " The optimal moisture content and the maximum dry density of 2:8 carbide-slag-solidified silt " change to, " The optimum moisture content and the maximum dry density of carbide-slag-solidified silt "

Response: It had been modified according to reviewer’s comment.

12. Response to comment: Page 8 lines 145 – 146, check that " According to the experience, the optimal moisture content of 2:8 carbide-slag-solidified soil was 13%-14%. " There is only one optimum moisture content or it is the range of optimum moisture content? Change " experience" to "experiment"

Response: " experience" had been changed to "experiment"

13. Response to comment: Change all "optimal moisture content" to "optimum moisture content"

All "optimal moisture content" had been changed to "optimum moisture content".

14. Response to comment: Page 8 line 153, "the effects on the optimal moisture content " change to "its effect on the optimum moisture content "

Response: It had been modified according to reviewer’s comment.

15. Response to comment: Page 8 line 154, " when making" change to " when preparing"

Response: It had been modified according to reviewer’s comment.

16. Response to comment: Page 9 line 167, " increasing age" change to " increasing curing time"

Response: It had been modified according to reviewer’s comment.

17. Response to comment: Page 10 line 192, " age" change to " curing time"

Response: It had been modified according to reviewer’s comment.

18. Response to comment: Page 11 line 218, "compacts the soil" change to " Densify the soil "

Response: It had been modified according to reviewer's comment.

19. Response to comment: Page 12 line 227, " affect the between" gelatin is not an appropriate meaning

Response: It had been modified according to reviewer's comment." Gelatin" had been changed to "Cementing Action"

20. Response to comment: Page 12 lines 229-230, " soil quality" what is the meaning of it?

Response: " soil quality" was not an appropriate meaning, it had been changed to "soil group".

21. Response to comment: Page 12 line 238-240, " the higher the fiber content, the more significant the compactness of the solidified soil. Moreover, the unconfined compressive strength was relatively high." the interpretation is unconvincing.

Response: We had re-written this part according to the Reviewer's suggestion.

22. Response to comment: Page 12 lines 289 to 290, The symbols of fiber intensity did not appear in the PDF format

Response: It had been modified according to reviewer's comment.

23. Response to comment: Page 16 lines 312, 322, 325 and 335, use more appropriate expression instead of like interweaving instead of " interleaving effects"

Response: It had been modified according to reviewer's comment. they had been changed to "intercrossing actions".

24. Response to comment: Page 17 lines 336, " suggests that" change to " revealed that"

Response: It had been modified according to reviewer's comment.

25. Response to comment: For figures, the axes title should be in the middle of the axes

Response: It had been modified according to reviewer’s comment.

Special thanks to you for your good comments.

We tried our best to improve the manuscript and made some changes in the manuscript. These changes will not influence the content and framework of the paper. And here we did not list the changes but marked in red in revised paper.

We appreciate for Editor and Reviewers' warm work earnestly, and hope that the correction will meet with approval. Once again, thank you very much for your comments and suggestions.
