# Supplementary material for: Experimental study on engineering properties of fiber-stabilized carbide-slag-solidified soil
Source: PLoS One. 2022 Apr 14;17(4):e0266732. doi: 10.1371/journal.pone.0266732 (PMC9009615; doi:10.1371/journal.pone.0266732)
Supplement: Attachment — Submitted filename: Comments to the author polus 28 1 2022.docx [file pone.0266732.s004.docx]

**Reviewer**

Manuscript Number: **PONE-D-22-00217**

Title: **Experimental study on engineering properties of fiber-stabilized carbide-slag-solidified soil**

This study aims to investigate the effect of fiber length and fiber content on the properties of carbide-slag-solidified soil based on unconfined compressive strength and indirect tensile strength tests.

The text is written clearly. The adopted methodology is appropriate. Some issues should be improved to make the article's to be clearer.

However, there are some comments for this study that need to be considered and implemented prior to the publication of this manuscript as follows. There are other comments in the PDF format of the manuscript.

1. Page 1 line 19, in abstract "The concepts of mass density "the density is mass per unit volume the expression "mass density" is an incorrect expression remove the mass
2. Page 1 line 19, in abstract "the root number of fibers " explain the root number? Is it just number of fibers?
3. All "Unconfined Compression Strength" change to 'unconfined compressive strength'
4. On Page #2 "Keywords: "Unconfined Compression Strength" change to " Unconfined Compressive Strength";
5. On Page #2 "Keywords: "mass density of fiber in solidified soil"; is a sentence not a keyword, change to " solidified soil ";
6. On Page #2 "Keywords: "root number of fiber in unit volume solidified soil", is a sentence not a keyword, it is suggested to be removed.
7. Page 3 line 50, "improve the compaction strength" change to " improve the compressive strength"
8. Page 3 line 51, " of cemented clay" is not clear is it clay stabilized with cement? Explain and correct it
9. Page 3 line 52, " the load cracking " is not an appropriate expression, correct it
10. Page 3 line 57, " interlacing mechanism " is not clear for me, explain that, several expressions used in the paper " interlacing, interweaving, interleaving, … what are the differences between them?
11. Page 6 line 111, " The optimal moisture content and the maximum dry density of 2:8 carbide-slag-solidified silt " change to, " The optimum moisture content and the maximum dry density of carbide-slag-solidified silt "
12. Page 8 lines 145 – 146, check that " According to the experience, the optimal moisture content of 2:8 carbide-slag-solidified soil was 13%-14%. " There is only one optimum moisture content or it is the range of optimum moisture content? Change " experience" to "experiment"
13. Change all "optimal moisture content" to "optimum moisture content"
14. Page 8 line 153, "the effects on the optimal moisture content " change to "its effect on the optimum moisture content "
15. Page 8 line 154, " when making" change to " when preparing"
16. Page 9 line 167, " increasing age" change to " increasing curing time"
17. Page 10 line 192, " age" change to " curing time"
18. Page 11 line 218, "compacts the soil" change to " Densify the soil "
19. Page 12 line 227, " affect the gelatin between" gelatin is not an appropriate meaning
20. Page 12 lines 229-230, " soil quality" what is the meaning of it?
21. Page 12 line 238-240, " the higher the fiber content, the more significant the compactness of the solidified soil. Moreover, the unconfined compressive strength was relatively high." the interpretation is unconvincing
22. Page 12 lines 289 to 290

The symbols of fiber intensity did not appear in the PDF format

23. Page 16 lines 312, 322, 325 and 335, use more appropriate expression instead of like interweaving instead of " interleaving effects"
24. Page 17 lines 336, " suggests that" change to " revealed that"
25. For figures, the axes title should be in the middle of the axes
