# Supplementary material for: Experimental study on engineering properties of fiber-stabilized carbide-slag-solidified soil
Source: PLoS One. 2022 Apr 14;17(4):e0266732. doi: 10.1371/journal.pone.0266732 (PMC9009615; doi:10.1371/journal.pone.0266732)
Supplement: Attachment — Submitted filename: Response to Reviewers.docx [file pone.0266732.s006.docx]

Dear Editor and Reviewers:

Thank you for your letter and for the reviewers’ comments concerning our manuscript entitled “Experimental study on engineering properties of fiber-stabilized carbide-slag-solidified soil” (ID: PONE-D-22-00217). Those comments are all valuable and very helpful for revising and improving our paper, as well as the important guiding significance to our researches. We have studied comments carefully and have made correction which we hope meet with approval. Revised portion are marked in red in the paper. The main corrections in the paper and the responds to the reviewer’s comments are as flowing:

Responds to the reviewer’s comments:

Reviewer #1:

1. Response to comment: The novelty and importance of the work should be stressed.

Response: It had been modified according to reviewer’s comment.

2. Response to comment: Table 1 and Fig. 1, standards used in these tests should be added and properly referenced.

Response: It had been modified according to reviewer’s comment.

3. Response to comment: Table 1: there are errors in the titles LL, PL…etc.

Response: The errors had been corrected。

4. Response to comment: Standards used in testing of the fiber (Table 3) should be added.

Response: It had been modified according to reviewer’s comment.

5. Response to comment: Quality of figures should be amended.

Response: It had been modified according to reviewer’s comment.

6. Response to comment: Style of Figures 5, 7, 9, 10 and 11. The titles of the axes should be centered, legends of the figures should be enhanced, and numbers should be more visible.

Response: It had been modified according to reviewer’s comment.

7. Response to comment: Justifications of the results should be added. The readers are more interested in the probable reasons of the expected behavior than only reporting the results.

Response: It had been modified according to reviewer’s comment.

Special thanks to you for your good comments.

Reviewer #2: 
1. Response to comment: Page 1 line 19, in abstract "The concepts of mass density "the density is mass per unit volume the expression "mass density" is an incorrect expression remove the mass.

Response: It had been modified according to reviewer’s comment.

2. Response to comment: Page 1 line 19, in abstract "the root number of fibers " explain the root number? Is it just number of fibers?

Response: Yes, it is just number of fibers, the root had been removed.

3. Response to comment: All "Unconfined Compression Strength" change to 'unconfined compressive strength'

Response: It had been modified according to reviewer’s comment.

4. Response to comment: On Page #2 "Keywords: "Unconfined Compression Strength" change to " Unconfined Compressive Strength";

Response: It had been modified according to reviewer’s comment.

5. Response to comment: On Page #2 "Keywords: "mass density of fiber in solidified soil"; is a sentence not a keyword, change to " solidified soil ";

Response: It had been modified according to reviewer’s comment.

6. Response to comment: On Page #2 "Keywords: "root number of fiber in unit volume solidified soil", is a sentence not a keyword, it is suggested to be removed.

Response: It had been modified according to reviewer’s comment.

7. Response to comment: Page 3 line 50, "improve the compaction strength" change to " improve the compressive strength"

Response: It had been modified according to reviewer’s comment.

8. Response to comment: Page 3 line 51, " of cemented clay" is not clear is it clay stabilized with cement? Explain and correct it

Response: Yes, it is clay stabilized with cement, it had been corrected to "cement-clay".

9. Response to comment: Page 3 line 52, " the load cracking " is not an appropriate expression, correct it

Response: It had been modified according to reviewer’s comment.

10. Response to comment: Page 3 line 57, " interlacing mechanism " is not clear for me, explain that, several expressions used in the paper " interlacing, interweaving, interleaving, … what are the differences between them?

Response: There is no difference between " interlacing, interweaving, interleaving ", they were uniformly corrected as “intercrossing”.

Intercrossing mechanism refers to the fact that there are countless intercrossing points of disordered fibers in the soil. If the fibers are subjected to force at the intersection of fibers, there is a trend of displacement, and other fibers will encounter to prevent this displacement. In other words, the stress and deformation of any segment of fiber will affect the fibers in all directions intertwined with it, thus forming the stress area of space. As shown in Figure 1.

Figure 1 Intercrossing mechanism

11. Response to comment: Page 6 line 111, " The optimal moisture content and the maximum dry density of 2:8 carbide-slag-solidified silt " change to, " The optimum moisture content and the maximum dry density of carbide-slag-solidified silt "

Response: It had been modified according to reviewer’s comment.

12. Response to comment: Page 8 lines 145 – 146, check that " According to the experience, the optimal moisture content of 2:8 carbide-slag-solidified soil was 13%-14%. " There is only one optimum moisture content or it is the range of optimum moisture content? Change " experience" to "experiment"

Response: " experience" had been changed to "experiment"

13. Response to comment: Change all "optimal moisture content" to "optimum moisture content"

All "optimal moisture content" had been changed to "optimum moisture content".

14. Response to comment: Page 8 line 153, "the effects on the optimal moisture content " change to "its effect on the optimum moisture content "

Response: It had been modified according to reviewer’s comment.

15. Response to comment: Page 8 line 154, " when making" change to " when preparing"

Response: It had been modified according to reviewer’s comment.

16. Response to comment: Page 9 line 167, " increasing age" change to " increasing curing time"

Response: It had been modified according to reviewer’s comment.

17. Response to comment: Page 10 line 192, " age" change to " curing time"

Response: It had been modified according to reviewer’s comment.

18. Response to comment: Page 11 line 218, "compacts the soil" change to " Densify the soil "

Response: It had been modified according to reviewer’s comment.

19. Response to comment: Page 12 line 227, " affect the between" gelatin is not an appropriate meaning

Response: It had been modified according to reviewer’s comment.” Gelatin” had been changed to “Cementing Action”

20. Response to comment: Page 12 lines 229-230, " soil quality" what is the meaning of it?

Response: " soil quality" was not an appropriate meaning, it had been changed to “soil group”.

21. Response to comment: Page 12 line 238-240, " the higher the fiber content, the more significant the compactness of the solidified soil. Moreover, the unconfined compressive strength was relatively high." the interpretation is unconvincing.

Response: We had re-written this part according to the Reviewer’s suggestion.

22. Response to comment: Page 12 lines 289 to 290, The symbols of fiber intensity did not appear in the PDF format

Response: It had been modified according to reviewer’s comment.

23. Response to comment: Page 16 lines 312, 322, 325 and 335, use more appropriate expression instead of like interweaving instead of " interleaving effects"

Response: It had been modified according to reviewer’s comment. they had been changed to “intercrossing actions”.

24. Response to comment: Page 17 lines 336, " suggests that" change to " revealed that"

Response: It had been modified according to reviewer’s comment.

25. Response to comment: For figures, the axes title should be in the middle of the axes

Response: It had been modified according to reviewer’s comment.

Special thanks to you for your good comments.

We tried our best to improve the manuscript and made some changes in the manuscript. These changes will not influence the content and framework of the paper. And here we did not list the changes but marked in red in revised paper.

We appreciate for Editor and Reviewers' warm work earnestly, and hope that the correction will meet with approval. Once again, thank you very much for your comments and suggestions.
