# Supplementary material for: Experimental study on engineering properties of fiber-stabilized carbide-slag-solidified soil
Source: PLoS One. 2022 Apr 14;17(4):e0266732. doi: 10.1371/journal.pone.0266732 (PMC9009615; doi:10.1371/journal.pone.0266732)
Supplement: Attachment — Submitted filename: Response to Reviewers.docx [file pone.0266732.s008.docx]

Dear Editor and Reviewers:

Thank you for your letter and for the reviewers’ comments concerning our manuscript entitled “Experimental study on engineering properties of fiber-stabilized carbide-slag-solidified soil” (ID: PONE-D-22-00217R1). Those comments are all valuable and very helpful for revising and improving our paper, as well as the important guiding significance to our researches. We have studied comments carefully and have made correction which we hope meet with approval. Revised portion are marked in red in the paper. The main corrections in the paper and the responds to the reviewer’s comments are as flowing:

Responds to the reviewer’s comments:

Reviewer #1:

We are deeply sorry for the mistake we made in the first revision! In this time, a marked-up copy of our manuscript that highlights changes made to the original version had been upload that as a separate file labeled 'Revised Manuscript with Track Changes'(from page 38 to 57 of PONE-D-22-00217_R2). At the same time, an unmarked version of our revised paper without tracked changes had been upload this as a separate file labeled 'Manuscript'(from page 9 to 28 of PONE-D-22-00217_R2). And the changes were also within the reply section. Special thanks to you for your good comments.

1. Response to comment: The novelty and importance of the work should be stressed.

Response: It had been modified according to reviewer’s comment.

Emphasize the novelty and importance of the work as follow in Page 3 line 59 of Manuscript (page 11 and 40 of PONE-D-22-00217_R2).

” In this study, the properties of fiber-carbide slag-solidified silt were studied according to fiber length and fiber content. The research findings are expected to serve as a reference for applying fiber-stabilized carbide-slag-solidified soil in road engineering. It can protect the environment, improve the resource utilization efficiency of carbide slag, reduce the project cost, achieve sustainable development, and achieve remarkable economic and social benefits.”

2. Response to comment: Table 1 and Fig. 1, standards used in these tests should be added and properly referenced.

Response: It had been modified according to reviewer’s comment.

Standards used in these tests had been added and properly referenced as follow in Page 4 line 67 of Manuscript (page 12 and 41 of PONE-D-22-00217_R2). ” According to Test Methods of soils for Highway Engineering (JTG 3430-2020) [20], the physical indexes and particle gradation of test soil are determined. The physical indexes are listed in Table 1, and the particle size distribution is shown in Fig 1,”

3. Response to comment: Table 1: there are errors in the titles LL, PL…etc.

Response: The errors had been corrected as follow in Page 4 line 77 of Manuscript (page 12 and 41 of PONE-D-22-00217_R2).

| **Property** | **ω_L_** | **ω_P_** | ***I*_P_** | ***G*** | **σ_dmax_** | ***OMC*** |
| --- | --- | --- | --- | --- | --- | --- |
|  | **%** | **%** | **%** |  | **g/cm^3^** | **%** |
| **Value** | 31.1 | 22.8 | 8.3 | 2.45 | 1.86 | 12.15 |

4. Response to comment: Standards used in testing of the fiber (Table 3) should be added.

Response: It had been modified according to reviewer’s comment. Standards used in these tests had been added and properly referenced as follow in Page 5 line 89 of Manuscript (page 13 and 42 of PONE-D-22-00217_R2). ”According to Testing Method for Length of Man-made Staple Fibers(GB/T 14336-2008) [21] and Testing Method for Tensile Properties of Man-made Staple Fibers(GB/T 14337-2008) [22]，the physical and mechanical properties of the fibers are listed in Table 3.”

5. Response to comment: Quality of figures should be amended.

Response: It had been modified according to reviewer’s comment. Quality

of figures had been amended as follow.

**Fig 1. Particle-size distribution of the soil.** Before the tests, the soil samples were crushed by soil milling and passed through a 2.36 mm square hole sieve. The soil collected under the sieve was used for testing. D60 of the soil is 1.208mm, D30 of the soil is 0.344mm, D10 of the soil is 0.093mm, $C_{\text{u}}$ and $C_{\text{c}}$ of the soil are 13 and 1.06.

**Fig 2. The carbide slag used in the study.** Because carbide slag contains a large amount of calcium hydroxide, it has strong hygroscopicity, so it needs to be dried before use. After drying and dehydration, it was crushed by soil milling and passed through a 0.075 mm square hole sieve.

**Fig 3. The fibers used for the study.** The fibers used for the study is polypropylene monofilament staple fibers，which have high strength and elastic modulus, excellent dispersion, no agglomeration, stable chemical properties, acid and alkali resistance.

**Fig 4. Prepared specimen of fiber stabilized carbide slag solidified soil.** The size of the specimens were 50 mm in diameter, 50 mm in height, and the degree of compaction was 96%.After the specimens were produced using the static pressure method, they were placed in a curing box (temperature = 20±2 °C; relative humidity ≥ 95%).

**Fig 5. Compaction Curve of Carbide Slag Solidified Soil.** According to the experiment, the optimum moisture content of 2:8 carbide-slag-solidified soil was 13.62 %, and the maximum dry density was 1.68g/cm^3^.

**Fig 6. Failure of the Unconfined Compressive Test specimen.** There are many and small cracks on the surface of the sample. When the failure of the sample is serious, no fracture surface has been formed, resulting in the sliding of the soil block. At the same time, obvious expansion occurs at the end of the sample.

**Fig 7. Unconfined Compressive Strength of fiber stabilized carbide slag solidified soil.** For the same fiber length and content, the unconfined compressive strength of the solidified soil significantly increased with increasing curing time. For each group of fibers, the 7 and 28 d unconfined compressive strength increased with increasing fiber content, but the degree of improvement was limited.

**Fig.8. Indirect Tensile Strength test specimen.** Under the action of static load, the failure of specimen shows three processes: initial crack generation, crack propagation, new crack generation and fiber pulling out. After cracking, crack development and fiber pulling out will be carried out simultaneously.

**Fig 9. Results of Indirect Tensile Strength Test.** For the same fiber length and content, the indirect tensile strength of the solidified soil significantly increased with increasing curing time. With an increase in the fiber length for the same fiber content, the indirect tensile strength of the 7 d solidified soil did not increase significantly; in contrast, the indirect tensile strength of the 28 d solidified soil increased significantly.

**Fig 10. Results of Unconfined Compressive Test with same *N*_f_.** Under the same conditions, the unconfined compressive strength of the solidified soil slightly increased with the fiber content. The unconfined compressive strength of the solidified soil reinforced with 19 mm fibers was the highest.

**Fig 11. Results of Indirect Tensile Test with same *N*_f_.** Under the same $N_{\text{f}}$ conditions, the indirect tensile strength of the 7d solidified soil increased with the fiber content. The indirect tensile strength of the solidified soil containing 19 mm long fibers was the highest. The 28d indirect tensile strength of each solidified soil increased significantly with the incorporation of fibers. The solidified soil containing 19 mm long fiber exhibited the highest indirect tensile strength.

6. Response to comment: Style of Figures 5, 7, 9, 10 and 11. The titles of the axes should be centered, legends of the figures should be enhanced, and numbers should be more visible.

Response: It had been modified according to reviewer’s comment. Style of Figures 5, 7, 9, 10 and 11. The titles of the axes had been centered, legends of the figures had been enhanced, and numbers had been more visible as follow.

**Fig 5. Compaction Curve of Carbide Slag Solidified Soil.** According to the experiment, the optimum moisture content of 2:8 carbide-slag-solidified soil was 13.62 %, and the maximum dry density was 1.68g/cm^3^.

**Fig 7. Unconfined Compressive Strength of fiber stabilized carbide slag solidified soil.** For the same fiber length and content, the unconfined compressive strength of the solidified soil significantly increased with increasing curing time. For each group of fibers, the 7 and 28 d unconfined compressive strength increased with increasing fiber content, but the degree of improvement was limited.

**Fig 9. Results of Indirect Tensile Strength Test.** For the same fiber length and content, the indirect tensile strength of the solidified soil significantly increased with increasing curing time. With an increase in the fiber length for the same fiber content, the indirect tensile strength of the 7 d solidified soil did not increase significantly; in contrast, the indirect tensile strength of the 28 d solidified soil increased significantly.

**Fig 10. Results of Unconfined Compressive Test with same *N*_f_.** Under the same conditions, the unconfined compressive strength of the solidified soil slightly increased with the fiber content. The unconfined compressive strength of the solidified soil reinforced with 19 mm fibers was the highest.

**Fig 11. Results of Indirect Tensile Test with same *N*_f_.** Under the same $N_{\text{f}}$ conditions, the indirect tensile strength of the 7d solidified soil increased with the fiber content. The indirect tensile strength of the solidified soil containing 19 mm long fibers was the highest. The 28d indirect tensile strength of each solidified soil increased significantly with the incorporation of fibers. The solidified soil containing 19 mm long fiber exhibited the highest indirect tensile strength.

7. Response to comment: Justifications of the results should be added. The readers are more interested in the probable reasons of the expected behavior than only reporting the results.

Response: It had been modified according to reviewer’s comment. Justifications of the results had been added as follow in Page 10 line 196 of Manuscript (page 18 and 47 of PONE-D-22-00217_R2).

”Because fibers are stable materials, they do not react with silt or calcium carbide slag, and their effect on soil is similar to the mechanism by which plant roots solidify in soil. According to the bending mechanism, the bending fibers between soil particles limit the change in the relative position of the soil particles through the pressure and frictional force on the concave side of the soil particles, stabilizing the soil. According to the intercrossing mechanism, the intercrossing fibers in soil form a spatial network structure, and the action of external forces in one direction is counteracted by the entire network structure. In addition, the incorporation of fibers effectively reduces the internal pores of solidified soil, densifies the soil, and improves its mechanical properties; this can be called the “filling mechanism.” Based on the bending, intercrossing, and filling mechanisms, the preconditions for fiber stability in the soil are that the fiber must have a sufficient length and minimum distribution density in the soil.”

Reviewer #2:

Response to comment: This study aims to investigate the effect of fiber length and fiber content on the properties of carbide-slag-solidified soil based on unconfined compressive strength and indirect tensile strength tests. The text is written clearly. The adopted methodology is appropriate. Some issues should be improved to make the article's to be clearer, the manuscript was highly improved, just some correction highlighted in the PDF file.

Response: Special thanks to you for your good comments.

some correction highlighted in the PDF file had been done as follow.

Page 2 line 30, "the only" had been instead of "an effective".

Page 2 line 41, "cement-clay" had been instead of "cement-clay mix".

Page 3 line 56, "root" had been removed.

Page 7 line 130, "13%-14%" had been instead of "13.5%".

Page 14 line 267, "" had been instead of "".

**Fig 1， “distribution the soil”** had been instead of “**distribution of the soil**”

We tried our best to improve the manuscript and made some changes in the manuscript. These changes will not influence the content and framework of the paper. And here we did not list the changes but marked in red in revised paper.

We appreciate for Editor and Reviewers' warm work earnestly, and hope that the correction will meet with approval. Once again, thank you very much for your comments and suggestions.
